# Lutetium-177 radiopharmaceuticals in cancer care: progress and challenges

**DOI:** 10.3389/fphar.2026.1907065

**Published:** 2026-09-07

**Authors:** Weirong Shi, Limin Zhang, Xinyue Feng, Jiayu Liu, Xinru Li, Xueming Dong, Andrea Li-Ann Wong, Xiaoguang Chen, Lingzhi Wang, Boon-Cher Goh

**Affiliations:** 1 Health Science Center, Yangtze University, Jingzhou, China; 2 Jingzhou Hospital of Traditional Chinese Medicine, Jingzhou, China; 3 The Third Clinical Medical College of Yangtze University, Jingzhou, China; 4 School of Biological Sciences, Nanyang Technological University, Singapore, Singapore; 5 Cancer Science Institute of Singapore, National University of Singapore, Singapore, Singapore; 6 Yong Loo Lin School of Medicine, NUS Center for Cancer Research (N2CR), National University of Singapore, Singapore, Singapore; 7 Department of Haematology-Oncology, National University Cancer Institute, Singapore, Singapore; 8 Department of Pharmacology, Yong Loo Lin School of Medicine, National University of Singapore, Singapore, Singapore

**Keywords:** cancer, combination therapy, Lutetium-177, radioimmunotherapy, radioligand therapy, targeted radioactive therapy

## Abstract

Lutetium-177 (^177^Lu)–based Radioligand Therapy (RLT) has emerged as an important modality in precision oncology, with demonstrated clinical benefit in selected malignancies. Its clinical relevance is underscored by regulatory approvals of Lutathera® for neuroendocrine tumours and Pluvicto® for metastatic castration-resistant prostate cancer. However, despite these advances, the field faces significant challenges that limit broader clinical translation and optimisation. In this review, we provide a critical and balanced evaluation of the current ^177^Lu RLT landscape, integrating evidence from clinical trials, emerging targets, and translational studies. We highlight that much of the existing evidence is derived from small, non-randomised studies with inherent risk of bias, limiting the strength of clinical conclusions. Key challenges include the lack of standardised and clinically actionable dosimetry, insufficient predictive biomarkers beyond target expression, vulnerability of isotope supply chains, and the development of drug-evasive traits in malignant cells. In addition, heterogeneity in trial design and limited long-term safety data further constrain clinical implementation. We propose a strategic roadmap to advance ^177^Lu RLT toward a more rigorous, reproducible, and equitable precision oncology framework, emphasising the transition from empiric application to evidence-based personalisation.

## 1 Introduction: the hype and the reality

Cancer constitutes a preeminent global health concern, characterized by enduringly elevated incidence and mortality rates that place a substantial strain on healthcare systems and society. 2022 global cancer statistics estimated approximately 20 million new cases and 9.7 million fatalities attributable to the disease (Bray et al., 2024). Beyond being a principal obstacle to extended life expectancy, cancer imposes a considerable socioeconomic burden, the magnitude of which is differentially distributed across various tumor types, distinct global regions, and patient genders (Chen et al., 2023). Although therapeutic strategies—particularly targeted therapies and immunotherapies—have led to transformative advances in the fight against cancer (Lloyd et al., 2024; Wang et al., 2024; Yang et al., 2023), significant clinical challenges remain, including the progression to late-stage metastatic disease, non-responsiveness to immunotherapy, and eventual relapse (Sgouros et al., 2020). Consequently, a novel therapeutic approach is urgently required—Radioligand Therapy (RLT).

RLT is gaining recognition as an innovative and well-tolerated targeted therapeutic modality demonstrating robust efficacy across a spectrum of cancers, exhibiting significant advantages in efficacy, safety, and compliance. Unlike radiotherapy, RLT’s radiation is not applied externally but is systemic or local delivery, similar to chemotherapy or biological targeted therapies. Cytotoxic radiation can be selectively deposited into either malignant neoplastic cells or the surrounding Tumor Microenvironment (TME). In contrast to certain biologic therapies that have failed due to the selected drug targeting an incorrect pathway (Lin et al., 2019), RLT has a much lower reliance on understanding signal pathways to block putative drivers of cancer phenotypes. In contrast to most conventional systemic oncological therapies, RLT exhibits a favorable therapeutic profile characterized by pronounced efficacy coupled with limited systemic toxicity (Gill et al., 2017).

**FIGURE 1:**
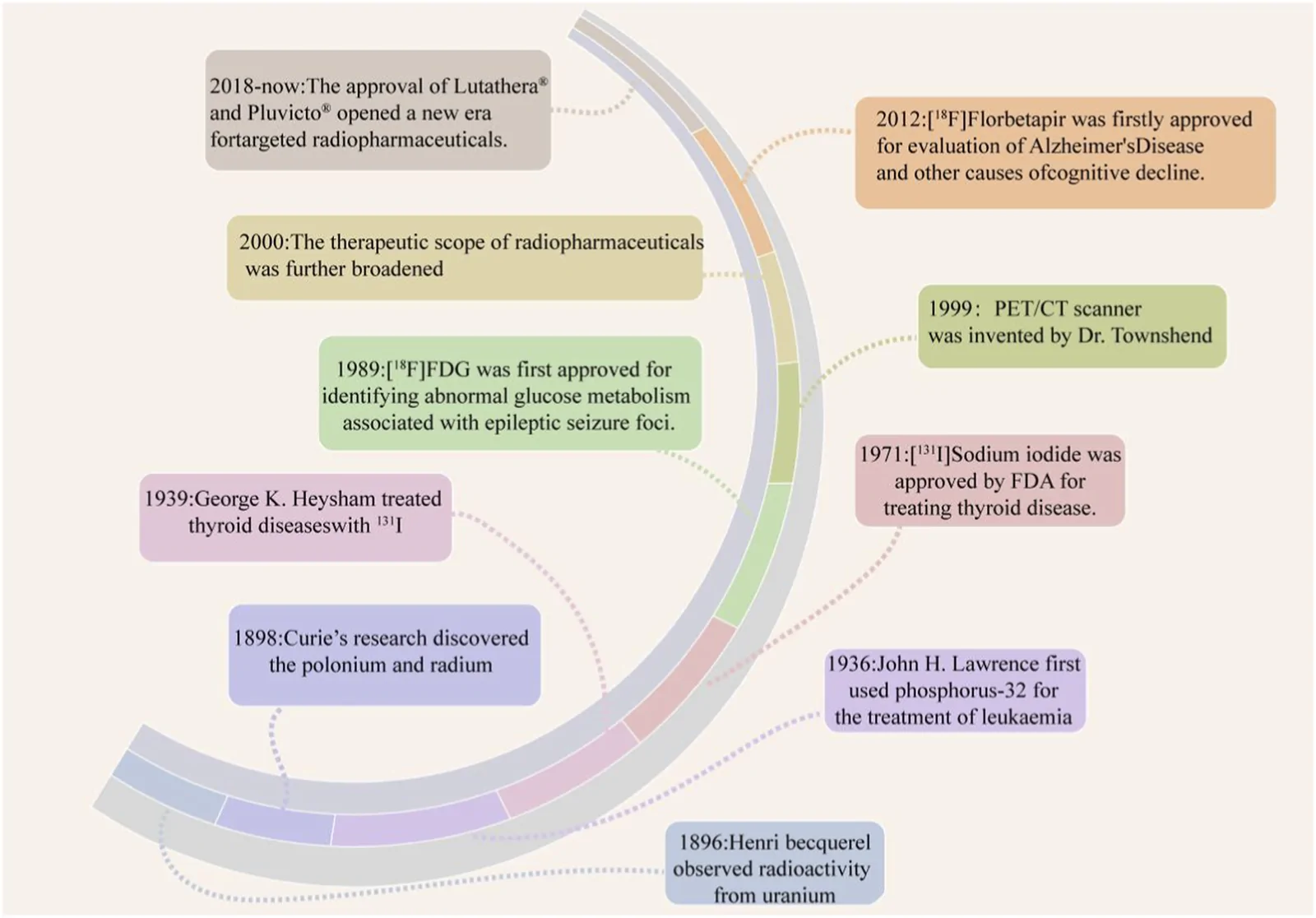
Chronology of radiopharmaceutical development: from the discovery of natural and artificial radionuclide to clinical practice.

The development of radiopharmaceuticals has undergone nearly a century of progress (Figure 1). In 1896, Henri Becquerel’s discovery facilitated the systematic study of radioactive processes and characterized the spontaneous penetrating rays emitted by uranium salts. This advancement revolutionized physics and established the foundation for subsequent medical applications (Torre et al., 2015). Becquerel’s findings inspired the Curies to conduct systematic investigations into radioactive elements, leading to the discovery of polonium and radium (Goldsmith, 2020). In the early 20th century, George K. Heysham pioneered the use of radioactive Iodine-131 (^131^I) for treating thyroid diseases (Costa et al., 2021). The advent of nuclear piles, particle acceleration devices, and chemical fractionation techniques has significantly simplified and enhanced the production of radioactive isotopes, greatly advancing the development of radiotherapy. A comprehensive review of the historical development of radiopharmaceuticals has recently been published. To avoid unnecessary duplication, the present review does not reproduce this chronology, and readers are referred to that publication for a detailed historical overview (Zhang S. et al., 2025).

**FIGURE 2:**
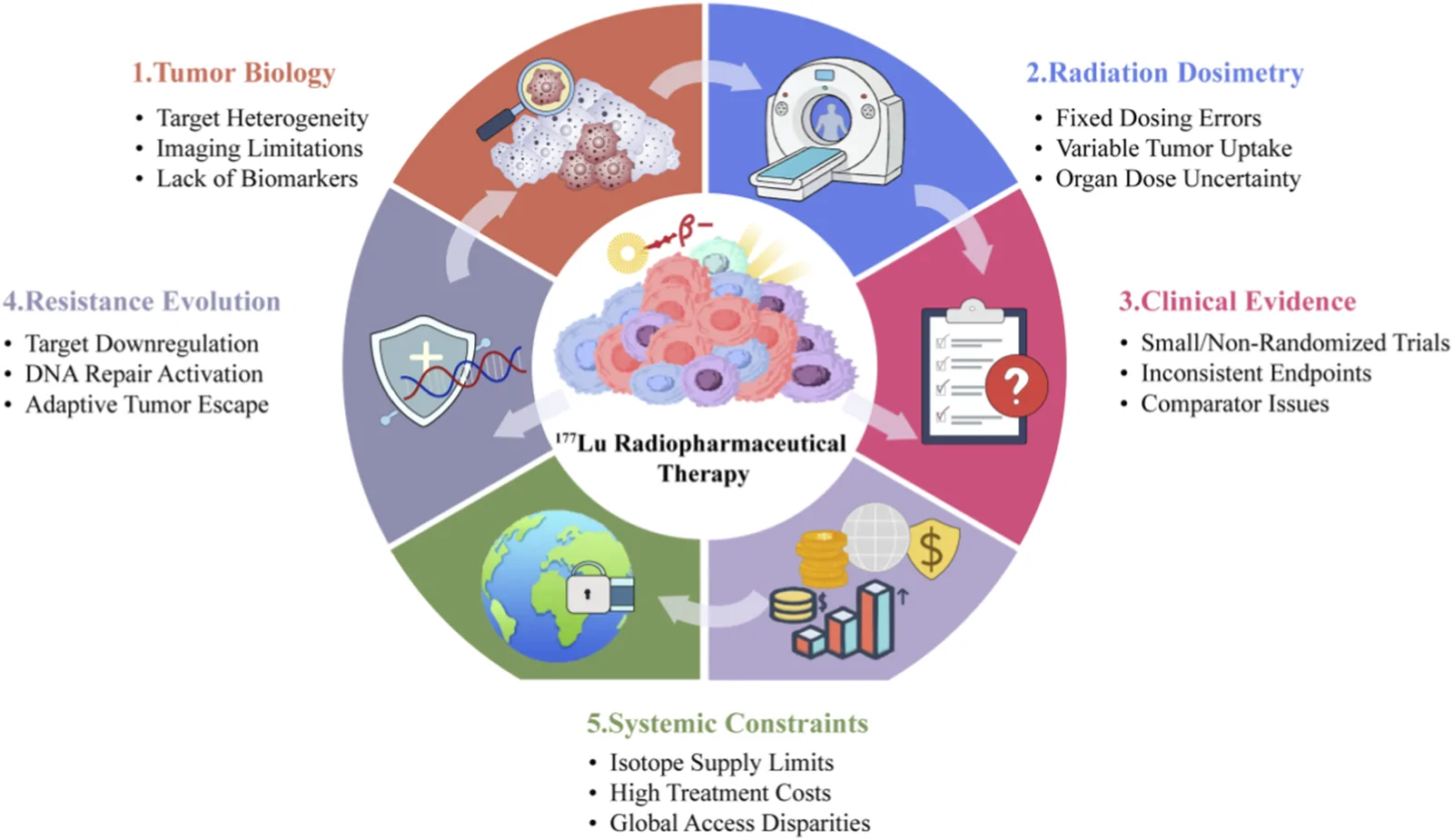
Major challenges facing the clinical translation of ^177^Lu radiopharmaceutical therapy. The five interconnected domains include tumor biology (heterogeneity, imaging limitations, and lack of predictive biomarkers), radiation dosimetry (fixed dosing, variable tumor uptake, and uncertainty in absorbed-dose estimation), clinical evidence (limited randomized evidence and heterogeneous endpoints), resistance evolution (target downregulation, DNA repair activation, and adaptive tumor escape), and systemic constraints (limited isotope supply, high treatment costs, and global disparities in access). Together, these challenges highlight key priorities for advancing precision radiopharmaceutical therapy through improved patient selection, individualized dosimetry, optimized clinical trial design, resistance-targeted strategies, and strengthened healthcare infrastructure.

A consistent upward trend in research articles addressing “cancer theranostics” has been observed over the past few years (Herrmann et al., 2020). Existing reviews related to radiopharmaceuticals often provide broad overviews of their development, clinical application, and associated challenges (Zhang S. et al., 2025). In the present review, we focus specifically on ^177^Lu-labeled radioligand therapy, given its remarkable success in phase III clinical trials. It begins by elucidating the fundamental concepts of radiopharmaceuticals and the developmental history of ^177^Lu-labeled agents, systematically reviewing their key carrier molecules and biological foundations. Subsequently, it emphasizes the ^177^Lu-labeled radioligands therapy in conbination with immunotherapy and targeted therapy regimens. The key innovation of this review is its clinically driven framework, which uses the outcomes of approved radiopharmaceuticals as the starting point to identify the critical design principles of successful carrier platforms through backward reasoning. These insights are then translated forward to guide future carrier development and rational combination strategies, while simultaneously informing improvements in clinical trial design and carrier synthesis. By linking carrier engineering, preclinical research, clinical validation, and therapeutic implementation within a unified translational framework, this review addresses a major limitation of previous reviews, which largely summarized evidence at individual stages without integrating the critical connections across the entire development pathway. We propose a conceptual framework encompassing five key bottlenecks that define the current state of the field: (i) tumour biology and target heterogeneity, (ii) lack of standardised and clinically integrated dosimetry, (iii) limitations in clinical evidence and trial design, (iv) emergence of treatment resistance, and (v) system-level barriers including isotope supply, infrastructure, and access (Figure 2). By structuring the discussion around these axes, we aim to provide a mechanistic and translational roadmap to guide the evolution of ^177^Lu RLT toward evidence-based precision oncology.

A targeted literature search was conducted between December 2025 and January 2026 using the electronic databases PubMed, Web of Science, and Google Scholar. Search terms included combinations of “^177^Lu,” “radiopharmaceutical therapy,” “radioligand therapy,” “targeted radionuclide therapy,” “cancer,” “dosimetry,” “clinical trial,” “precision oncology,” and “combination therapy.” Boolean operators (AND and OR) were used to optimize search sensitivity and specificity across databases. In addition, relevant regulatory guidelines, policy documents, and institutional reports were identified through targeted searches of publications from regulatory agencies, including the U.S. Food and Drug Administration (FDA) and the European Medicines Agency (EMA). Priority was given to peer-reviewed articles published between 2020 and 2025 to capture recent advances in radiopharmaceutical therapy, clinical pharmacology, precision oncology, dosimetry, regulatory science, and healthcare implementation. Seminal publications published before 2020 were also included where appropriate to provide historical context and establish the conceptual and scientific framework of the field.

## 2 Approved agents: a closer look at the evidence

### 2.1 Definition and classification of radiopharmaceuticals

**FIGURE 3:**
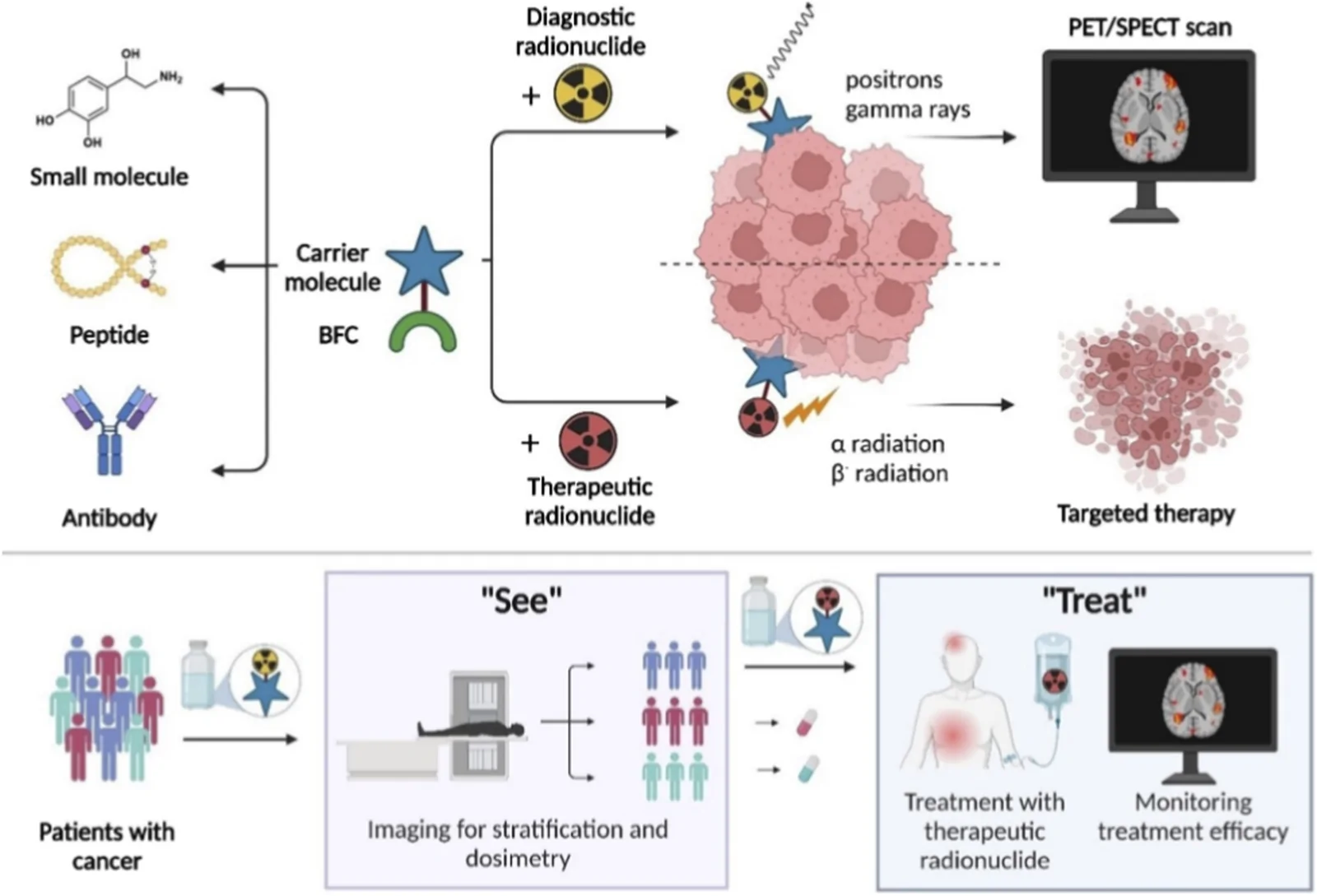
Principles of radiotheranostics: see what you treat and treat what you see. A radiotheranostic agent contains a carrier molecule targeting a tumor-specific receptor or antigen labeled with a diagnostic radionuclide or a therapeutic radionuclide with or without a linker. Diagnostic radiotracers emit positrons or gamma rays and allow for tumor visualization, patient stratification, and therapeutic dosimetry through positron emission tomography (PET) or single photon emission computed tomography (SPECT) imaging. Therapeutic radiotracers tag and kill cancer cells via lethal α or β− radiation. BFC: bifunctional chelating agent. Radiotheranostic landscape: A review of clinical and preclinical development. (Tran et al., 2025) Eur J Nucl Med Mol Imaging. Vol. 52/7; 2025, reproduced with permission from SNCSC.

Radiopharmaceuticals constitute a class of theranostic molecular probes that utilize targeted carriers, such as small molecules, peptides, or antibodies, to specifically deliver radionuclides to lesion cells (Bollineni et al., 2014). When integrated with imaging modalities like Single Photon Emission Computed Tomography (SPECT) or Positron Emission Tomography (PET), they enable non-invasive, precise visualization of systemic pathologies and stratification of cancer patients (Figure 3). Their fundamental structure comprises a carrier-linker-radionuclide assembly. The carrier acts as an anchor, facilitating localized accumulation of the radionuclide within or near cancerous tissues (Herrmann et al., 2020). The high-energy emissions from the radionuclide within target cells can induce DNA single-strand or double-strand breaks, directly mediating cytotoxic effects on cancer cells (Bolcaen et al., 2021). The linker serves as a critical bridge between the carrier and radionuclide; an optimized linker enhances the *in vivo* stability of the radiopharmaceutical, preventing premature radionuclide dissociation and consequent off-target radiation damage to non-organs, the radiation-induced bystander effect (Aboagye et al., 2023).

### 2.2 The development of Lutetium-177 radiopharmaceuticals

Early clinical investigations commenced in 1960, when Anderson et al. first reported the administration of ^177^Lu in chloride- or citrate-stabilized injectable forms as a therapeutic option for patients with plasma cell myeloma. Although therapeutic outcomes were suboptimal, this pioneering work established the initial safety profile of this radionuclide for human applications (Anderson et al., 1960). Over the subsequent 3 decades, research progress remained limited until 1991, when the Schlom group successfully developed ^177^Lu-labeled monoclonal antibody CC49, marking the systematic investigation of ^177^Lu as a targeted therapeutic vector (Schlom et al., 1991). With the advent of Peptide Receptor Radionuclide Therapy (PRRT) concepts in the 21st century, targeted agents such as ^177^Lu-DOTA-TATE achieved critical breakthroughs. Theirs demonstrated effectiveness and tolerability in patients with Neuroendocrine Tumors (NETs) spurred large-scale multicenter clinical trials, culminating in Food and Drug Administration (FDA) approval in 2018 as the first PRRT agent in this domain. The NETTER 1 trial established PFS benefit for midgut NETs progressing on somatostatin analogues (Pavel et al., 2025). The VISION trial was a well designed phase III study that met its primary endpoints, showing improvements in Overall Survival (OS) and radiographic Progression Free Survival (rPFS) (Ruder et al., 2025). Based on these trial results, Pluvicto® and its companion imaging agent [^68^Ga]Ga-PSMA-11 received FDA and European Medicines Agency (EMA) approvals for treating Metastatic Castration-Resistant Prostate Cancer (mCRPC) in 2022 (Gandhi et al., 2025). Additionally, ^177^Lu-labeled monoclonal antibodies, including J591 and Rituximab, have advanced from preclinical evaluation to phase I/II clinical trials for indications such as advanced prostate cancer and non-Hodgkin lymphoma, thereby expanding their clinical utility (Shim et al., 2023).

Although numerous β-emitting radionuclides have been investigated as potential therapeutic isotopes (Champion et al., 2016), their clinical translation has been constrained by limited isotope availability, complex radiochemistry, and challenges associated with large-scale production and radiolabeling. In contrast, ^177^Lu has emerged as the most widely adopted therapeutic radionuclide because of its favorable physical and chemical properties. It emits medium-energy β-particles (E_max = 0.497 MeV) that provide an effective tissue penetration range (approximately 1–2 mm), enabling localized tumor irradiation while minimizing collateral damage to surrounding normal tissues. Simultaneously, its low-energy γ-photons (113 and 208 keV) are ideally suited for SPECT imaging, allowing quantitative assessment of biodistribution, treatment response, and patient-specific dosimetry. Furthermore, its physical half-life of 6.65 days closely matches the pharmacokinetics of peptides and monoclonal antibodies, facilitating both prolonged tumor retention and efficient systemic clearance. These characteristics make ^177^Lu particularly well suited for theranostic applications, in which the same radionuclide can be used for molecular imaging, patient selection, dosimetry, treatment planning, and subsequent radionuclide therapy (Das and Banerjee, 2016).

The differences between ^177^Lu and α-emitting radionuclides arise primarily from their distinct radiophysical characteristics, which fundamentally determine their biological effects, toxicity profiles, dosimetry, and clinical applications. As a low-linear energy transfer (LET) β-emitter, ^177^Lu predominantly induces DNA damage indirectly through the generation of reactive oxygen species, resulting mainly in single-strand DNA breaks and repairable double-strand breaks. Consequently, its cytotoxic effects are distributed over a relatively longer particle range, producing a beneficial cross-fire effect that is particularly advantageous for heterogeneous tumors. In contrast, α-particles possess an extremely high LET (>50–100 keV/μm) and an ultrashort tissue range (<100 μm), depositing large amounts of energy within a few cell diameters. This results in dense clusters of complex DNA double-strand breaks that are difficult to repair and largely independent of oxygen availability or cell-cycle status, thereby producing substantially greater relative biological effectiveness (RBE) (Lacerda et al., 2025).

Although α-particle therapy theoretically offers greater tumor specificity because of its short path length, this advantage may be offset by radionuclide recoil following α-decay. The recoil energy can dissociate radioactive daughter nuclides from the targeting vector, permitting their redistribution to normal organs—including the kidneys, salivary glands, liver, and bone marrow—and thereby increasing the risk of off-target toxicity (Song et al., 2025). By comparison, the dosimetry of ^177^Lu is considerably more mature because its accompanying γ-emissions permit serial quantitative SPECT/CT imaging for individualized absorbed-dose estimation. In contrast, the absence of suitable imaging emissions from most α-emitters makes quantitative dosimetry substantially more challenging and currently limits widespread implementation of personalized dose optimization (Akhtar et al., 2012).

These radiobiological differences also influence clinical application. ^177^Lu radiopharmaceuticals are generally preferred for patients with advanced malignancies who have moderate-to-high tumor burden and require an optimal balance between efficacy, safety, and individualized dosimetry. Conversely, α-emitting radiopharmaceuticals may be particularly advantageous for patients who have developed resistance to β-emitting therapies, have relapsed following ^177^Lu treatment, or harbor minimal residual disease or disseminated micrometastases, where their high cytotoxic potency and short particle range may provide superior tumor control while limiting irradiation of surrounding normal tissues. Nevertheless, direct comparative clinical evidence remains limited, and the optimal selection between β- and α-emitting radionuclides will likely depend on tumor biology, disease burden, target expression, and patient-specific dosimetric considerations.

## 3 Carriers of Lutetium-177 radiopharmaceuticals

The carrier serves as the guiding core that enables precise radionuclide therapy with ^177^Lu radiopharmaceuticals, directly determining therapeutic efficacy (Altunay et al., 2021). In ^177^Lu-labeled agents, the carrier fulfills two critical functions. First, it provides targeted delivery: by carrying the radioactive therapeutic moiety and leveraging its own high affinity for disease-specific targets, the carrier directs the active component to the intended tissue site, ensuring therapeutic specificity (Kim et al., 2025). Second, it ensures radionuclide stability: through covalently attached chelators (e.g., DOTA, DTPA), the carrier firmly complexes ^177^Lu, preventing its dissociation during systemic circulation. This maintains effective retention of the radionuclide within the target lesion while minimizing off-target radiation exposure (Price and Orvig, 2014). By modulating the *in vivo* biological behavior of the radiopharmaceutical, the carrier enhances tissue penetration, accelerates blood clearance, and reduces immunogenicity, thereby optimizing overall pharmacokinetic properties to improve therapeutic outcomes and mitigate adverse effects (Altmann et al., 2021). The ultimate goal is to achieve a synergistic balance among target affinity, pharmacokinetic profile, and therapeutic index.

**FIGURE 4:**
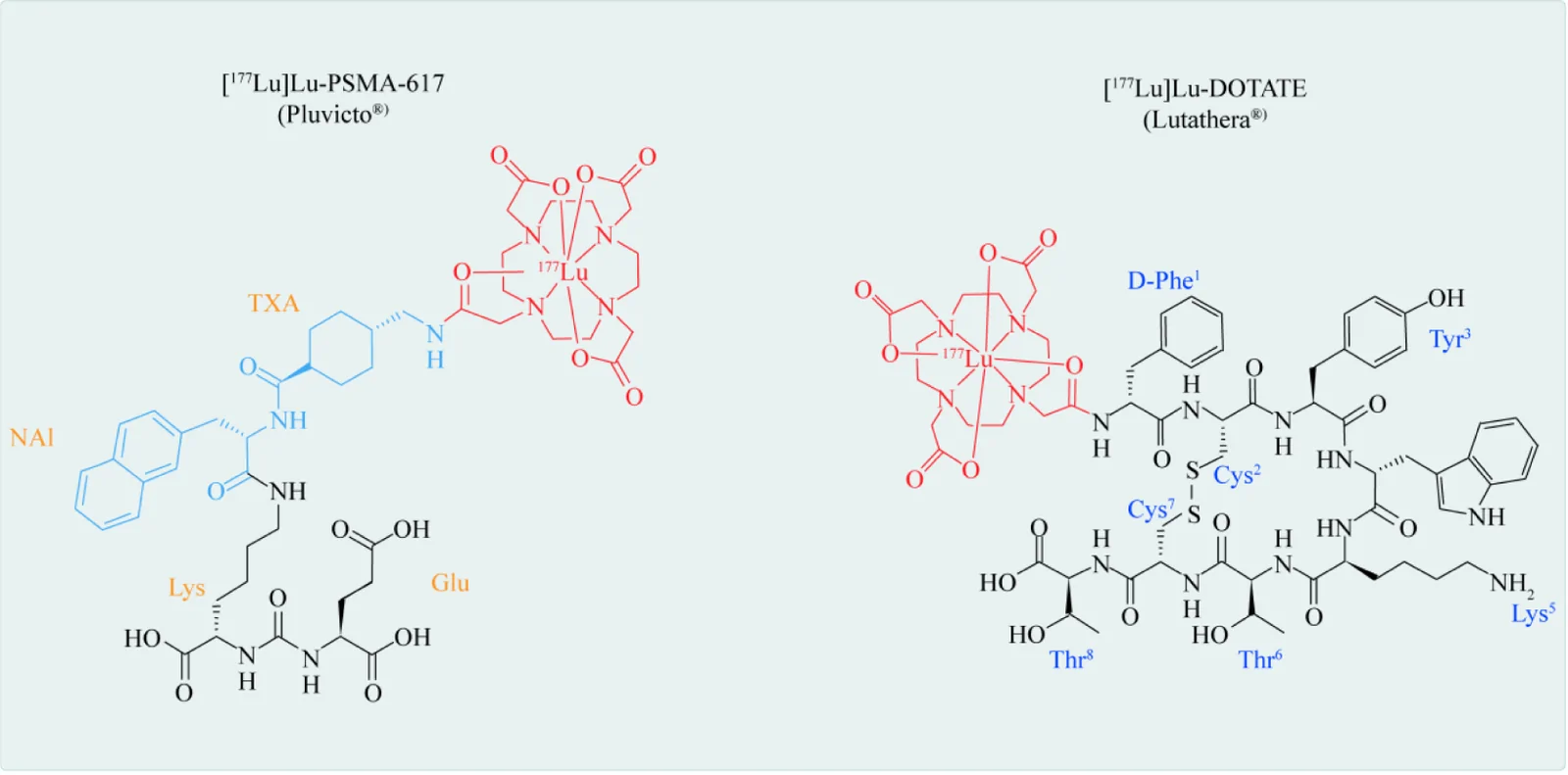
The molecular structures of Pluvicto® (^177^Lu-PSMA-617) and Lutathera® (^177^Lu-DOTA-TATE).

In recent years, ^177^Lu-PSMA-617 and ^177^Lu-DOTA-TATE have gained significant recognition as key radiopharmaceuticals. Their superior targeting affinity, robust *in vivo* stability, and marked clinical efficacy position them as successful paradigms in carrier design. This section will focus on these two representative agents, summarizing both their successful attributes and inherent limitations. Drawing on these foundations, we will then systematically introduce emerging carrier platforms designed to overcome these constraints, highlighting their distinct advantages. Figure 4 illustrates the molecular structures of these two compounds.

### 3.1 Small molecule peptide-based carrier: ^177^Lu-DOTA-TATE

^177^Lu-DOTA-TATE’s carrier, DOTA-TATE, functions as a somatostatin analog that exhibits high affinity for Somatostatin Receptor 2 (SSTR2) (O'Neill et al., 2020). Its core structure comprises two components: the somatostatin analog octapeptide (TATE) as the targeting moiety and the macrocyclic compound DOTA as the stable chelator, which are covalently linked (Zhang S. et al., 2025). DOTA (1,4,7,10-tetraazacyclododecane-1,4,7,10-tetraacetic acid) is a dual-function chelating agent that can chelate assorted radionuclides with an octadentate coordination geometry. The mechanism of action is based on the TATE peptide acting as a high-affinity agonist for SSTR2, enabling specific recognition and binding to SSTR2, which is commonly overexpressed on the surface of neuroendocrine tumor cells. Upon binding, this typically initiates the internalization of the receptor-ligand complex, thereby delivering the therapeutic radionuclide ^177^Lu into the tumor cells for close-range, high-efficacy internal radiation therapy (Hennrich and Eder, 2022). Direct conjugation of DOTA to the N-terminus of the peptide may result in a relatively low molecular weight during renal excretion, facilitating filtration through the renal tubules and rapid clearance. To effectively manage potential nephrotoxicity, appropriate measures, such as co-infusion of amino acids, are recommended (Giussani et al., 2012). A study involving two tumor-bearing rat models reported that a decrease in long-term nephrotoxicity was observed after administering of high doses of ^177^Lu-DOTA-TATE combined with lysine co-injection. The conclusion indicates that fractionated dosing with lysine co-injection contributes to mitigating renal injury (Zellmer et al., 2020). Compared to DOTA-TOC, another somatostatin analog, DOTA-TATE exhibits an affinity for SSTR2 that is 8–9 times higher (Reubi et al., 2000). Currently, widely used SSTR analogs are all agonists of somatostatin receptors, meaning they activate the receptor, ultimately promoting internalization, trapping of the radionuclide, and release of radiation to induce cell death (Bodei and Weber, 2018).

### 3.2 Small molecule inhibitors-based carrier: ^177^Lu-PSMA-617

PSMA-617 represents a highly selective, small-molecule inhibitor engineered to bind specifically to PSMA—a type II transmembrane glycoprotein (∼100 kDa) that serves as a potential therapeutic target in prostate oncology because of its overexpression in prostate tumor tissues (Patell et al., 2023). PSMA exhibits elevated expression levels in prostate cancer and particularly upregulates in low differentiation, metastatic, and metastasis disease forms (Silver et al., 1997). Structurally, PSMA-617 integrates the PSMA-binding motif Glu-NH-CO-NH-Lys, linked via a spacer incorporating the amino acids 2-naphthyl-l-alanine (Nal) and Tranexamic Acid (TXA) to the macrocyclic chelator DOTA (Benešová et al., 2015). The introduced spacer enhances metabolic stability while modulating *in vivo* biodistribution (Evans et al., 2020). Simultaneously, the linker functions as a critical bridge between the chelating moiety and the pharmacophore, maintaining high receptor-binding affinity while mitigating steric hindrance (Brandt et al., 2018). Fine-tuning the linker’s chemical architecture—including considerations of size, conformation, hydrophilicity, stability, and relative molecular mass—allows for the induction of multifaceted physicochemical and pharmacokinetic outcomes that significantly elevate the overall performance profile of the radiopharmaceutical agent (Li et al., 2020). The preparation of the PSMA-617 ligand employs solid-phase peptide synthesis methodologies, as documented in earlier studies (Eder et al., 2012). Relative to conventional small-molecule peptides, this class of agents demonstrates enhanced tissue penetration capacity, superior pharmacokinetic behavior, and more pronounced affinity and specificity for the target (Altunay et al., 2025). These attributes commonly facilitate higher target-to-nontarget uptake ratios—a critical determinant for both molecular imaging quality and the efficient deposition of therapeutic radiation dose (Zechmann et al., 2014).

### 3.3 Next-generation carriers

Although small molecule/small peptide carriers remain the cornerstone of the field of ^177^Lu-labeled radiopharmaceuticals due to their maturity, they are not specific to FDA-approved tumor indications. Their application can be extended to other malignant tumor classification, (e.g., breast carcinoma, colorectal carcinoma, and pancreatic cancer) where recent evidence has confirmed aberrantly high expression of various receptors (Reubi et al., 2005). Consequently, numerous novel carriers for ^177^Lu radiopharmaceuticals remain to be explored, which serve not as replacements but as complements and expansions of their therapeutic scope. A range of emerging carrier platforms provides solutions for refractory tumors, heterogeneous tumors, and combination therapies. The field continues to advance rapidly toward diversification in carrier structures, expansion of target systems, and intelligent functional design.

Antibodies and their derivatives represent a crucial category within radiotherapeutic delivery platforms. Table 1 provides a comparative overview of their properties with small molecule and peptide carriers. The evolution of antibody-based radiotherapy has been propelled by the progressive integration of immunotherapy into oncology, continuous refinements in antibody engineering, and advances within the Antibody Drug Conjugate (ADC) domain (Sgouros et al., 2020). Bispecific Antibodies (BsAbs) constitute a novel antibody class capable of engaging two distinct epitopes simultaneously, offering unique mechanisms of action. Compared to monospecific antibodies, they demonstrate superior tumor targeting and enhanced inhibitory effects (Klein et al., 2024). ON105 is a ^177^Lu-labeled BsAb targeting both oxidized macrophage Migration Inhibitory Factor (oxMIF) and a Histamine Succinyl Glycine (HSG) hapten for directing radiolabeled constructs to oxMIF-expressing tumors, currently under investigation in murine colorectal and pancreatic xenograft models. Recent preclinical data from single-dose ON105 treatment in CT26 and CFPAC-1 models indicate significant tumor regression persisting for 22 days (Puchol Tarazona et al., 2024). Nevertheless, a significant constraint associated with full-length antibodies is their substantial molecular mass, which impedes tissue penetration and delays systemic elimination. The biological half-life of an IgG1 antibody typically ranges between 14 and 21 days (Moek et al., 2017), substantially exceeding the physical half-life of most clinically relevant radionuclides. Consequently, a significant proportion of the radionuclide decays while the antibody remains within the circulatory system, leading to unnecessary irradiation of radiosensitive tissues. This has directed attention towards immunoglobulin-derived antigen-binding fragments with accelerated clearance kinetics (White et al., 2021). Antigen-binding fragments offer advantages over intact antibodies, including lower immunogenicity, enhanced tissue infiltration, facile synthesis, and capacity for Blood Brain Barrier (BBB) traversal (Rashid, 2022). Their smaller size also entails limitations, most notably rapid degradation, elevated renal uptake, and a shorter half-life, which may constrain therapeutic applications (Kashihara et al., 2025). For radiotherapy, antibody fragments are often conjugated to proteins such as albumin or undergo PEGylation to extend their circulating half-life (Piramoon et al., 2021).

**TABLE 1:** A comparative analysis:small-molecule,peptide and antibodies and their derivatives.

Feature dimension	Small-molecule	Peptide	Antibodies and their derivatives
Molecular weight	Very small (<1 kDa)	Small (1–3 kDa)	Large (6–150 kDa)
Targeting affinity	High	Moderate	Very high
Pharmacokinetics (blood half-life)	Very fast (minutes–hours)	Fast (hours)	Slow (days–weeks)
Depth of tumor invasion	Apable of penetrating the entire tumor tissue	Rapidly and extensively	With restricted distribution primarily around blood vessels.
Advantages	Simple synthesis, Extremely high permeability	Excellent tissue penetration, Low immunogenicity, Ease of chemical modification	High target specificity, Prolonged circulation time, Mature clinical validation
Challenges	Rapid clearance, Off-target risk	Poor *in vivo* stability, High renal uptake	Poor *in vivo* stability, High renal uptake

Although agents directed against SSTR and PSMA continue to predominate in contemporary therapeutic clinical investigations, the landscape of targeted radiopharmaceuticals is rapidly expanding, a trend substantiated by emerging clinical data and a broadening array of preclinical candidates. Targeting Fibroblast Activation Protein (FAP)—a protein highly expressed in over 90% of epithelial cancer-associated fibroblasts—facilitates a direct attack on the tumor microenvironment, surmounts tumor cell heterogeneity, and exhibits broad-spectrum efficacy across multiple solid tumors, including pancreatic, lung, and breast cancers (Bellavia et al., 2022). Carbonic Anhydrase IX (CAIX), a transmembrane isoenzyme, manifests a constrained distribution within normal physiological tissues, yet is consistently overexpressed across the majority of solid tumor malignancies (Becker, 2020). CAIX interacts with pH regulators, lowering extracellular pH, promoting a reverse pH differential in the TME, and elevating intracellular pH (Venkateswaran and Dedhar, 2020). Extracellular acidification promotes tumor progression through the elimination of neighboring host cells, disruption of cell-matrix adhesion, and suppression of immune responses; conversely, intracellular alkalinization facilitates cell proliferation and migration while concurrently inhibiting apoptosis. Four trials involve CAIX-targeting agents (NCT05868174, NCT05663710, NCT05239533, NCT07197580), all utilizing ^177^Lu labels. Table 2 summarizes ^177^Lu radiopharmaceuticals with clinical relevance.

**TABLE 2:** Comprehensive comparison summary table of ^177^Lu radiopharmaceuticals.

Radiotheraputic agents	Target	Carrier type	Indications	Therapeutic effect	Adverse events (Grade 3 or higher)	References
^177^Lu-DOTATATE	SSTR	Small molecule	GEP-NETs	ORR: 18% mPFS: 22.9 months mOS: 48.4 months	Myelosuppression Gastrointestinal reactions	Mittra (2018)
^177^Lu-PSMA-617	PSMA	Small molecule	mCRPC	ORR: 29.8% mPFS: 8.7 months mOS: 15.3 months	Myelosuppression Xerostomia/dysgeusia	Kwan et al. (2025)
^177^Lu-FAPI-46	FAP	Small molecule	Advanced solid tumors	ORR: 38% mPFS: 6.2 months mOS: 13.8 months	Mild myelosuppression Mild gastrointestinal reactions	Ghahraman et al. (2025)
^177^Lu-trastuzumab	Her 2	Humanized mAb	Advanced breast cancer	ORR: 35% mPFS: 6.2 months mOS: 13.8 months	Cardiotoxicity Myelotoxicity Infusion reaction	Abbas et al. (2013)
^177^Lu-tetulomab	EGFR	Humanized mAb	Advanced NSCLC	ORR: 22% mPFS: 4.8 months mOS: 10.2 months	Acne-like rash Mild myelosuppression Rare interstitial pneumonia	Zakaly et al. (2020)
^177^Lu-rituximab	CD 20	Full-length mAbs	Relapsed B-cell non-Hodgkin lymphoma	ORR: 58% mPFS: 14.2 months	myelosuppression Infusion reaction Rare HBV reactivation	Fu et al. (2023)
^177^Lu-DOTA-FRα	FRα	Small molecule	Ovarian cancer	ORR: 38% mPFS: 6.8 months mOS: 14.5 months	Mild myelosuppression Mild Gastrointestinal reactions Rare oral mucositis	Guleria et al. (2018)
[^177^Lu]Lu-DOTA-hu3F8	GD2	Humanized mAb	High-risk neuroblastoma	ORR: 42% 2-year event-free survival rate: 48%	Myelosuppression Neuralgia Rare allergic reactions	Kramer et al. (2021)
^177^Lu-EB-FAPI	FAP	Small molecule	mRAIR-TC	ORR: 25% DCR: 83%	Grade 3 and 4 hematologic toxicity	Müller et al. (2013)
^177^Lu-SibuDAB	PSMA	Small molecule	Prostate cancer	ADC: 9.9 ± 5.4 Gy/GBq	Myelosuppression	Ritt et al. (2025)
^177^Lu-PSMA-ALB-56	PSMA	Small molecule	Prostate cancer	ADC: 6.64 ± 6.92 Gy/GBq	—	Zheng et al. (2025)
^177^Lu-FAP-2286	FAP	Small molecule	Gastrointestinal tumours (FAP+)	DCR: 28.6%	Transient nausea, vomiting, and diarrhea	Bai et al. (2025)
^177^Lu-DPI-4452	CAIX	Peptide	Colorectal cancer Clear cell renal cell carcinoma	—	Renal AD:0.438 Gy/GBq	Fu et al. (2025)
^177^Lu-LNC1004	FAP	Small molecule	Progressive metastatic malignancies	ADC: 4.69 ± 3.83 Gy/GBq DCR: 46%	Hematotoxicity	Hou et al. (2025)
^177^Lu-J591	PSMA	Humanized mAb	Prostate cancer	Radiation-absorbed dose: 2.10 ± 0.60 mGy	—	Massière et al. (2024)
^177^Lu-LNC1011	PSMA	Small molecule	mCRPC	ASED: 0.18 mSv/MBq	Hematotoxicity	Vallabhajosula et al. (2005)
^177^Lu-edotreotide	SSTR2	Peptide	GEP-NETs	PFS: 27.5 months mPFS: 23.9 months	TRAEs: 82% AE (3–4):18%	Walter et al. (2026)

**FIGURE 5:**
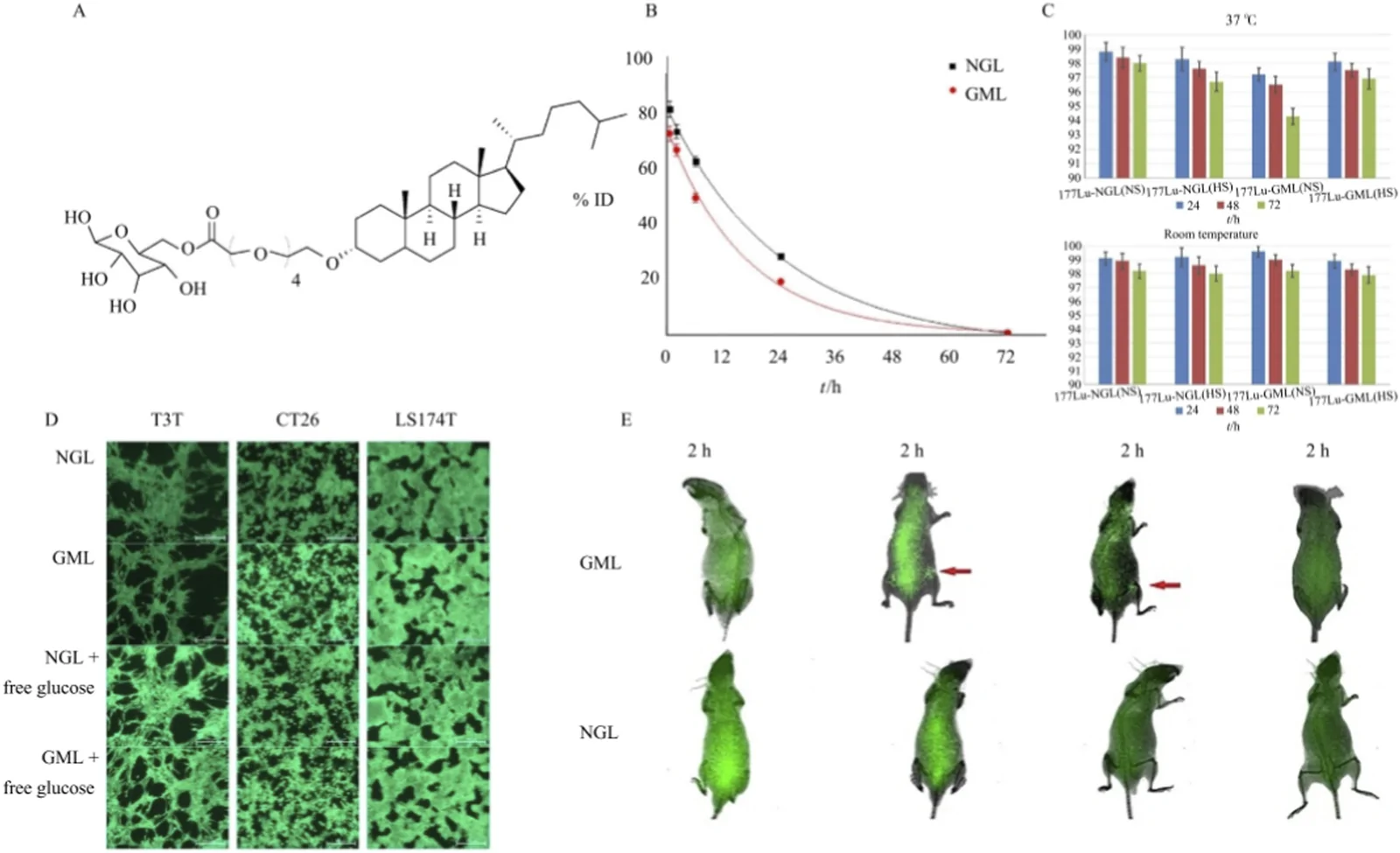
Glucose-modified liposomes (GMLs) increased the drug delivery to tumor cells. **(A)** Structural of Glycosyl derivative of cholesterol-L. **(B)** Blood clearance profile of ^177^Lu-NGL and ^177^Lu-GML. **(C)** Fluorescence microscopy images of NGL/GML in different cells (10 min incubation). **(D)** Imaging studies on LS174T tumor-bearing NOD/SCID mice after intravenous injection of ^177^Lu-GML and ^177^Lu-NGL (red arrows indicate the position of LS174T cancer sites). **(E)** Imaging studies on LS174T tumor-bearing NOD/SCID mice after intravenous injection of ^177^Lu-GML and ^177^Lu-NGL (red arrows indicate the position of LS174T cancer sites). Reprinted from Chinese journal of natural medicines, Vol. 23/6, (Wang et al., 2025). Advancements and applications in radiopharmaceutical therapy, Pages No. 9, Copyright (2025), with permission from Elsevier.

Liposomes are widely regarded as promising drug delivery carriers due to their low toxicity (Zhou et al., 2024). However, there are several challenges that they apply in clinical broadly, including improving tumor targeting, optimizing controlled drug release and extending systemic circulation time. To address these issues, Cvjetinovic et al. (Cvjetinović et al., 2021) developed Glucose-Modified Liposomes (GMLs) based on the unique metabolic characteristics of tumor cells, particularly their elevated energy consumption and glucose utilization. Further studies utilized ^177^Lu-labeled GMLs, employing a straightforward and reliable radiotracer technique to demonstrate their significant potential for tumor accumulation in murine models bearing CT26 and LS174T tumors (Figure 5) However, the field is generating a variety of novel targets and ligands, each presented in small, flashy proof of concept studies. The vast majority will never progress to phase III.

## 4 Combination therapy with Lutetium-177 radiopharmaceuticals

**FIGURE 6:**
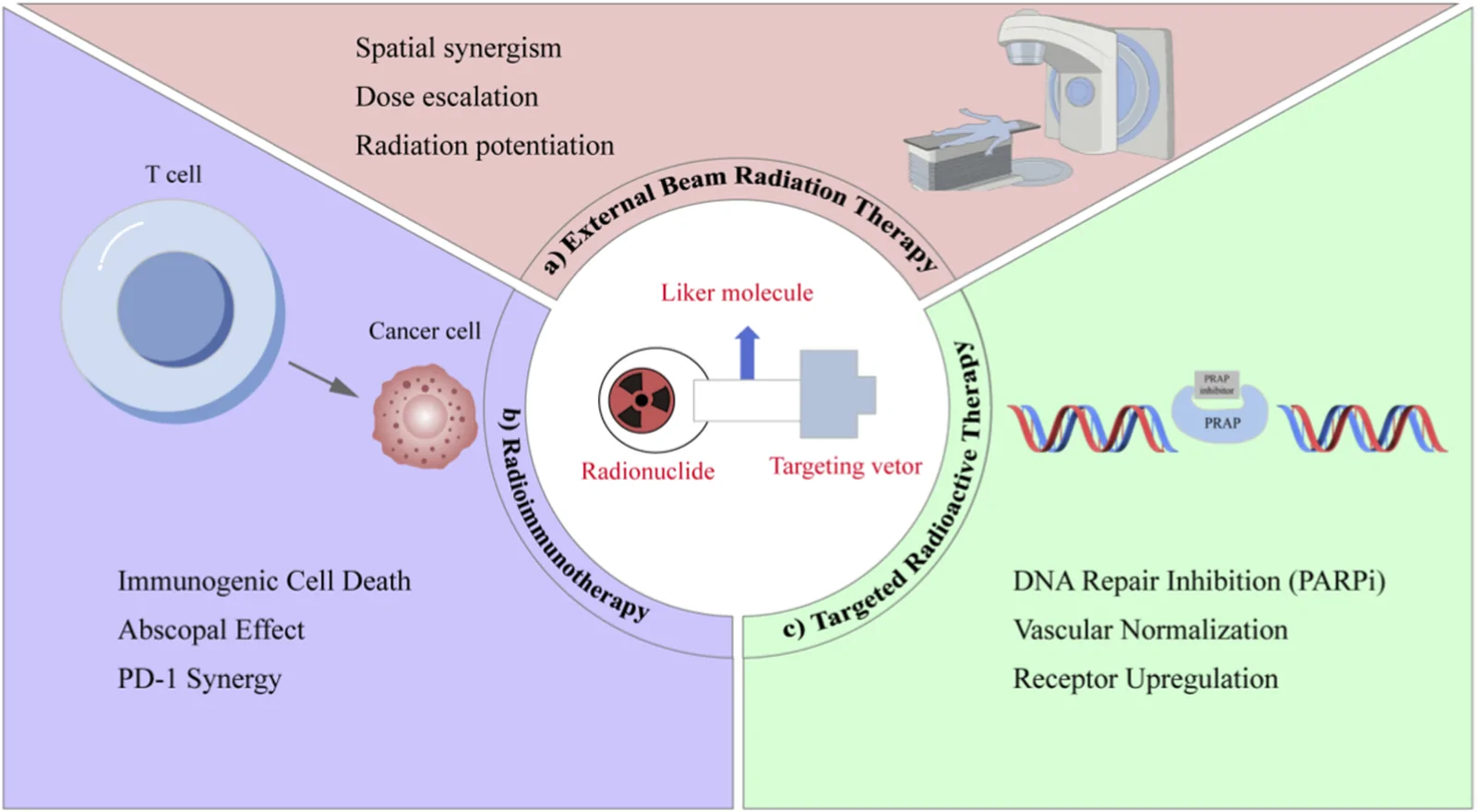
Schematic illustration of the principal therapeutic strategies for enhancing the efficacy of ^177^Lu radiopharmaceutical therapy (RPT) through rational combination approaches. A typical radiopharmaceutical consists of a targeting vector, radionuclide, and, where appropriate, a linker molecule, enabling selective delivery of ionizing radiation to tumor cells. Three complementary combination strategies are highlighted. **(a)** External beam radiotherapy (EBRT): combining RPT with EBRT may achieve spatially complementary irradiation, facilitate dose escalation to resistant lesions, and enhance local tumor control through radiation potentiation. **(b)** Radioimmunotherapy: integration with immunotherapy can stimulate immunogenic cell death, promote the abscopal effect, and augment antitumor immune responses, thereby improving the efficacy of immune checkpoint blockade (e.g., PD-1/PD-L1 inhibitors). **(c)** Targeted radioactive therapy: combining RPT with molecularly targeted agents, such as poly (ADP-ribose) polymerase (PARP) inhibitors, can impair DNA damage repair, enhance radiosensitivity, normalize the tumor vasculature, and increase target receptor expression, thereby improving radionuclide uptake and therapeutic efficacy. Collectively, these multimodal strategies aim to overcome treatment resistance, improve tumor control, and expand the therapeutic potential of precision radiopharmaceutical therapy.

While ^177^Lu-DOTA-TATE and ^177^Lu-PSMA-617 have demonstrated paradigm-shifting efficacy in neuroendocrine tumors and prostate cancer respectively, monotherapy regimens continue to grapple with the intractable challenges of acquired resistance, suboptimal response rates—particularly in patients with high tumor burden—and incomplete remissions (Urso et al., 2023). Combination therapeutic strategies have consequently emerged as a cornerstone for overcoming these limitations. This section will comprehensively delineate the rationales and emerging clinical evidence for combining ^177^Lu-labeled radiopharmaceuticals with external beam radiotherapy, immunotherapy, and molecularly targeted agents. Figure 6 is schematic overview of combinatorial approaches integrating ^177^Lu-labeled radiopharmaceuticals with external beam radiotherapy, immunotherapy, and targeted therapy in cancer treatment.

### 4.1 External beam radiation therapy (EBRT)

EBRT represents a standard approach in clinical oncology, utilizing high-energy rays such as X-radiation, protons, or electron particle beams targeting malignancies from outside the body to induce DNA damage in cancer cells, thereby inhibiting their replication and proliferation (Dudzinski et al., 2025). When combined with radiopharmaceutical agents, this synergistic strategy allows for precise ablation of tumor cells internally by targeted radiopharmaceuticals, while EBRT concurrently delivers effective doses to the tumor periphery. Such a combinatorial regimen significantly enhances overall therapeutic outcomes while attenuating risks of recurrence and metastasis (Abdel-Wahab and Giammarile, 2026).

Combined Peptide Receptor Radionuclide Therapy (PRRT) and EBRT represent a promising strategy for recurrent or progressive advanced meningiomas, warranting further investigation. In Kreissl et al.’s study (Kreissl et al., 2012), 10 patients with unresectable meningioma received PRRT using ^177^Lu-DOTA^0^, Tyr^3^-octreotate or -DOTA^0^, Tyr^3^-octreotide, followed by EBRT. Post-PRRT EBRT was delivered via Intensity-Modulated Radiotherapy (IMRT) over 5–6 weeks (median dose: 53.0 Gy). All patients underwent baseline morphological and PET assessment, with repeat imaging at 3–6 months post-treatment. Toxicities were graded per CTCAE v4.0. The combined regimen was feasible and well-tolerated, with favorable outcomes (8 patients achieving morphological stability, 1 partial response, and 1 complete response) sustained at a median follow-up of 12.8 months. These results exhibit strong congruence with the findings reported by Bartolomei et al. (2009).

### 4.2 Radioimmunotherapy

Radioimmunotherapy (RIT) represents a targeted cancer treatment strategy that leverages the conjugation of radionuclides with monoclonal antibodies (Schnog et al., 2024). The modality’s primary advantage stems from the antibody’s ability to selectively target tumor-associated antigens, thereby enabling the systemic delivery of cytotoxic radiation directly to malignant sites (White et al., 2021). Despite close to 40 years of R&D in RIT, its real-world implementation faces major bottlenecks. The approach fundamentally relies on the precise molecular recognition of tumor-specific antigens for the targeted delivery of therapeutic radionuclides. Ongoing research is investigating a diverse array of antibody-based constructs to serve as optimal carriers for RIT. These include full-length monoclonal antibodies, smaller antibody fragments (Jain et al., 2007) (such as F (ab′)_2_ or Fab), and novel engineered fusion proteins (Tsai and Wu, 2018) (e.g., single-chain variable fragments, scFv-Fc fusions, minibodies, bispecific antibodies, or nanobodies). Strategic selection of a specific monoclonal Antibody (mAb) format is pivotal for refining the Therapeutic Index (TI), a metric defined as maximizing tumor dose uptake while minimizing off-target toxicological damage to normal non-tumor tissues. A foundational study underscores the pivotal role of the Fc domain in full-length monoclonal antibodies. For instance, the full-length hu11B6 antibody, labeled with either ^225^Ac or ^177^Lu, exhibits specific binding to catalytically active human Kallikrein 2 (hK2) (Timmermand et al., 2019; Bicak et al., 2020; McDevitt et al., 2018), an antigen expressed in prostate cancer. Upon complex formation, the hu11B6:hK2 complex is internalized via Fc Receptor (FcRn)-mediated endocytosis. The subsequent transport to lysosomes for degradation facilitates the close proximity of the delivered therapeutic radionuclide to the cell nucleus, resulting in potent cytotoxic efficacy (Bicak et al., 2020).

RLT are increasingly recognized as a promising therapeutic modality, leveraging radioactive isotopes to target and eliminate tumor cells. Within this field, RIT specifically utilizes mAbs conjugated to radioisotopes, delivering cytotoxic radiation directly to antigen-expressing cancer cells while sparing healthy tissue. A pivotal advancement in lymphoid malignancies was the FDA approval of two RIT monoclonal antibodies for relapsed or refractory non-Hodgkin lymphoma: ^90^Y-ibritumomab tiuxetan (Zevalin®) in 2002 and ^131^I-tositumomab (Bexxar®) in 2003 (Witzig et al., 2002). Recent translational efforts have expanded the RIT paradigm to solid tumors, including Small Cell Lung Cancer (SCLC). The pretargeting Radioimmunotherapy (pRIT), which decouples antibody delivery from radioisotope administration to enhance tumor-to-background ratios, is also under investigation. A recent study in HER2-positive breast cancer models demonstrated that an anti-HER2-DOTA-based pRIT strategy combined with ^177^Lu-DOTA-Bn effectively suppressed tumor growth, improved survival, and exhibited minimal off-target toxicity (Cheal et al., 2018). These collective findings underscore the evolving and versatile application of radioimmunotherapy across diverse oncological indications.

The increasing number of RIT drugs in the preclinical pipeline is evident, yet significant challenges remain. Recent advances in phage surface display methodology, chemical reaction engineering and chemoenzymatic synthesis engineering, and radiolabeling strategies have solved many of these problem, yielding promising preclinical research outcomes (Lapi et al., 2024; Rondon et al., 2021). In summary, advancements in RIT drug development hold the potential for substantial benefit, aligning with our objective of delivering personalized, precision medicine for cancer therapy (White et al., 2021).

### 4.3 Targeted radioactive therapy

Targeted Radionuclide Therapy (TRT) is a therapeutic method that employs molecular carriers to direct a payload of radionuclides to malignant cells. It integrates the molecular targeting accuracy of molecularly targeted drug with the potent cytotoxic activity of radiotherapy to enable selective delivery of tumoricidal radiation to malignant cells (Primac et al., 2025). This mechanism of action, distinct from conventional approaches like systemic chemotherapy, EBRT and surgery, enables an unprecedented level of precision and adaptability. The benefits of this targeted approach extend beyond efficacy to include aspects related to Quality of Life (QoL), such as reduced toxicity, improved treatment adherence, and simplified dosing regimens (Fizazi et al., 2023). Optimal targets for TRT must meet strict predefined criteria to balance therapeutic efficacy, safety and clinical feasibility. Most are cell-surface proteins accessible to circulating radioactive pharmaceutical agents, and must be druggable, highly specific, have a good safety profile, and remain stable under pathophysiological conditions (Wang et al., 2023). While traditional therapeutic strategies have often prioritized targets based on their biological function within the disease process, the prevailing trend increasingly favors focusing on those essential elements—core pathways indispensable to tumorigenesis—rather than targets of mere convenience (Stock et al., 2015). In contrast, TRT does not require the target molecule to be a direct participant in oncogenesis. The paramount consideration is its stable and highly selective overexpression in tumor tissues, coupled with minimal to absent expression in normal tissues. For instance, PSMA exemplifies an ideal target; its expression is highly specific to prostate cancer, virtually absent in non-prostatic tissues, and further upregulated following Androgen Deprivation Therapy (ADT), offering a distinct therapeutic advantage. Conversely, targeting Receptor Tyrosine Kinases (RTKs) can present challenges, as their signaling networks are prone to compensatory rewiring by tumor cells, potentially undermining therapeutic efficacy (Tomuleasa et al., 2024).

Targeted Radioligand Therapy represents a promising novel therapeutic modality across multiple cancer types. In this context, Fibroblast Activation Protein (FAP)-targeted TRT has demonstrated acceptable tolerability and initial treatment response in patients with advanced cancers in preclinical studies and individual clinical case reports (Privé et al., 2023). A dosimetry study by Kuyumcu et al. (2021) investigated ^177^Lu-FAPI-04, administering a low dose (267.5 ± 8.6 MBq) to four patients with metastatic advanced cancer (breast, thymic, thyroid, and ovarian, respectively). Results indicated a low radiation absorbed dose to healthy high-risk organs. Tumor site doses were consistently higher than those to normal organs across bone, lymph gland, liver, and soft tissue metastases, underscoring a favorable therapeutic index. Proteolysis-Targeting Chimeras (PROTACs) constitute an advanced strategy for targeted protein degradation in cancer therapy. Zhang Y. et al. (2025) developed a dual-targeting, single-molecule theranostic probe (e.g., the radioactive ^177^Lu-P-A and its cold counterpart ^nat^Lu-P-A) for disease-activatable PROTACs, combined with Targeted Radionuclide Therapy, providing a potential approach for high specificity and efficacy against prostate cancer. The results demonstrate that the co-administration of ^nat^Lu/^177^Lu-P-A, integrating TRT and PROTACs, enhances radiosensitivity and promotes tumor cell apoptosis compared to monotherapies, highlighting a potent synergistic effect. Notably, under equivalent radioactive dosing, this combination therapy outperforms the commercially available agent ^177^Lu-PSMA-617, suggesting the potential to reduce radiopharmaceutical dose and associated toxicity without compromising efficacy. This combined strategy is critical and necessary for minimizing radionuclide dosage while preserving therapeutic outcomes (Zhang Y. et al., 2025).

The integration of TRT with diagnostic modalities solidifies its role as a precise medicine approach, offering clear advantages in oncology. Its success lies not only in broadening target scopes to include pan-cancer biomarkers for expanded patient cohorts, but also in delivering effective therapeutic options for specific cancers. From initial achievements with radioiodine for thyroid cancer and lymphoma (Sgouros et al., 2020; Bodei et al., 2022), to the presently granted regulatory approvals for NETs and prostate cancer as evidenced by net-1 and the VISION trial outcomes (Strosberg et al., 2017; Sartor et al., 2021), TRT continuously refines modern cancer therapy. Moving forward, researchers must deepen their understanding of the biological complexity underlying novel targets to optimally integrate TRT into comprehensive cancer care frameworks (Primac et al., 2025).

## 5 Clinical trials of Lutetium-177 radiopharmaceuticals in cancer

To evaluate the current status of ^177^Lu radiopharmaceuticals in clinical oncology, we conducted a literature survey via records retrieved from the ClinicalTrials.gov database. The search identified 106 registered clinical trials.

Analysis of the target population reveals NETs and mCRPC as the two fields with the most robust clinical evidence. NET-focused research predominantly employs somatostatin analogues such as ^177^Lu-DOTATATE/-TOC (e.g., NCT04375267), while mCRPC studies concentrate on ^177^Lu-PSMA-617 and its derivatives (e.g., NCT05613894). A significant emerging trend is the expansion of ^177^Lu-labeled radioligand therapy into Fibroblast Activation Protein (FAP)-positive solid tumors (e.g., NCT05723640, NCT06197139), HER2-positive breast cancer (e.g., NCT06824155, NCT07081555), and other malignancies, including pancreatic cancer, gliomas, and thyroid cancer. This demonstrates the increasing integration of ^177^Lu technology with a growing repertoire of molecular targets, with the goal of extending the precision efficacy of Radioligand Therapy to a broader spectrum of cancer types.

In endpoint selection throughout drug development, safety remains the paramount consideration across all phases. Across every stage—from early to late—key safety metrics consistently include adverse event rates and dose-limiting toxicities, especially cumulative absorbed doses to key organs such as skeletal marrow and kidneys (e.g., NCT06305962), reflecting the heightened emphasis on safety in radiopharmaceutical development. For efficacy assessments, earlier studies tend to rely on exploratory measures, such as Prostate Specific Antigen (PSA) response rates or Objective Response Rates (ORR), whereas later-phase trials transition to more rigorous endpoints like rPFS and OS. Notable is the growing focus in advanced-stage studies on QoL evaluations and patient-reported outcomes (e.g., FACT-P and FACT-RNT scales in NCT06517719), underscoring an evolving shift in oncology from tumor-centric to patient-centric metrics.

Regarding drug administration strategies, while empirical fixed-dose regimens are widely adopted in clinical practice, it remains unclear whether dosimetry-based personalized dosing can further optimize the therapeutic index. For instance, the phase III trial NCT05387603 is directly comparing personalized versus non-personalized ^177^Lu-DOTATOC regimens, and its results are anticipated to provide pivotal data for optimizing therapeutic protocols. Concurrently, evidence concerning the synergistic mechanisms and optimal timing of combination therapies remains limited. Although combination treatments represent a major research thrust, the optimal drug pairings, administration sequences, and dose-modification strategies are not yet established.

The clinical evidence supporting ^177^Lu-based radiopharmaceutical therapy remains subject to several important limitations, including patient selection, imaging assessment, dosimetry, and duration of follow-up. Broad eligibility criteria and the absence of standardized target-expression thresholds may dilute treatment effects by enrolling biologically heterogeneous populations. Likewise, variability in response assessment—including the use of different imaging criteria (e.g., RECIST, PCWG3, and PET-based criteria) and inconsistent assessment time points—complicates comparisons across studies and may obscure the true magnitude of antitumor activity. Furthermore, many studies have relied on administered activity rather than individualized absorbed dose estimates, limiting validation of radiobiological dose–response relationships. Finally, relatively short follow-up periods (typically <18 months) restrict the assessment of long-term overall survival, durability of response, and late treatment-related toxicities, thereby weakening the interpretation of surrogate endpoints such as progression-free survival. Collectively, these limitations reduce the comparability of clinical trials, hinder robust efficacy attribution, and impede definitive evaluation of long-term clinical benefit. Future confirmatory studies should prospectively incorporate biomarker-driven patient selection, standardized imaging protocols with independent central review, patient-specific three-dimensional dosimetry (including metrics such as D_mean and D_98), and extended follow-up of at least 5 years. These measures will facilitate the generation of more robust, comparable, and clinically meaningful evidence to support the continued development and regulatory evaluation of ^177^Lu radiopharmaceuticals. In summary, Supplementary Table S1 illustrates that the clinical development of ^177^Lu radiopharmaceuticals has entered a phase of rapid diversification, with increasing exploration of novel targets, combination strategies, and dosimetry-oriented trial designs. However, this expansion is accompanied by substantial structural weaknesses, including overrepresentation of early-phase small-cohort studies, limited randomized evidence, heterogeneous endpoints, and insufficient biological stratification. The field is therefore progressing faster in breadth than in evidentiary depth. Future progress will depend less on generating additional ligands and more on prioritising the most biologically credible targets, integrating dosimetry into treatment decision-making, embedding translational biomarker programs, and conducting comparative trials capable of defining true clinical value.

## 6 Conclusion and future perspectives

The clinical translation of ^177^Lu–labelled radiopharmaceuticals marks a major advance in precision oncology. However, their broader impact remains constrained by five interrelated bottlenecks spanning tumour biology, dosimetry, clinical evidence, resistance, and system-level implementation. These five bottlenecks define the current limitations of ^177^Lu RLT and highlight the need for a paradigm shift from empiric application to mechanism-driven, evidence-based precision therapy. Addressing these challenges in a coordinated manner is essential to fully realise the therapeutic potential of RLT.

First, tumour biology and target heterogeneity fundamentally limit treatment efficacy (Zhu et al., 2026). To overcome this, future strategies should incorporate multi-modal biomarker frameworks, integrating molecular imaging with genomic and transcriptomic profiling, as well as liquid biopsy approaches, to enable more accurate patient selection and real-time therapy response tracking.

Beyond the traditional reliance on target receptor expression, biomarker development for ^177^Lu radiopharmaceutical therapy is increasingly focused on multidimensional molecular profiling to identify tumors that are intrinsically susceptible to β-particle irradiation. Integrating genomic, transcriptomic, imaging, and circulating biomarkers offers the potential to improve patient selection, predict therapeutic response, identify resistance mechanisms, and enable adaptive treatment strategies. At the molecular level, predictive biomarkers extend beyond target expression to include tumor-intrinsic DNA damage response (DDR) capacity. In particular, genomic and transcriptomic signatures reflecting homologous recombination deficiency (HRD) or alterations in non-homologous end joining (NHEJ) may identify tumors with impaired DNA repair and increased radiosensitivity. Additional biomarkers associated with cell-cycle regulation, replication stress, and genomic instability may further refine prediction of treatment response. Functional imaging represents another critical component of precision radiopharmaceutical therapy. Radiomic analysis of pre-treatment PET/CT using ^68^Ga- or ^18^F-labeled tracers can quantify spatial tumor heterogeneity beyond conventional parameters such as SUV_max and SUV_mean. Higher-order radiomic features, including entropy, uniformity, gray-level co-occurrence matrix (GLCM), and gray-level run-length matrix (GLRLM) metrics, have shown promise for predicting absorbed-dose heterogeneity, treatment response, and early therapeutic resistance, although prospective multicenter validation remains essential. Liquid biopsy provides a complementary, minimally invasive approach for longitudinal disease monitoring. Dynamic assessment of circulating tumor DNA (ctDNA), circulating tumor cells (CTCs), extracellular vesicles, and circulating immune mediators may facilitate early response evaluation, detection of emerging resistance, and real-time assessment of treatment efficacy throughout the course of radiopharmaceutical therapy.

Despite these advances, the clinical implementation of multi-omics biomarkers remains limited by insufficient prospective validation, assay standardization, reproducibility across analytical platforms, and the absence of clinically validated decision thresholds. Patient-derived organoids (PDOs) offer a promising translational platform to address these challenges. By integrating *ex vivo* radiation-response profiling with matched genomic, radiomic, and liquid biopsy data, PDOs can functionally validate candidate biomarkers and establish biologically meaningful correlations between molecular characteristics and therapeutic response. Such an integrated framework could accelerate biomarker qualification, optimize patient stratification, and ultimately enable biologically informed, individualized dosing strategies for ^177^Lu radiopharmaceutical therapy. Second, the lack of standardised and clinically actionable dosimetry remains a central barrier to precision RLT. Current reliance on fixed-activity regimens fails to account for the substantial inter-patient variability in absorbed radiation dose, which can differ by up to 3–9 fold (Sjögreen Gleisner et al., 2022). This imprecise delivery strategy may lead to toxicity due to overexposure, or it may result in insufficient doses that compromise therapeutic efficacy. A critical translational consideration lies in the trade-off between fixed-activity regimens and dosimetry-guided dosing. Fixed dosing, as employed in VISION/NETTER-1, offers operational simplicity and multicenter feasibility, yet disregards profound inter-patient biokinetic variability, risking undertreatment in rapid clearers or excess toxicity in slow clearers. Dosimetry-guided approaches, utilizing serial imaging to constrain organ-at-risk doses (kidney ≤ 23 Gy, bone marrow ≤ 2 Gy) and maximize tumor exposure (≥80 Gy for optimal response), provide superior biological individualization but demand substantial infrastructure and expertise. We advocate a risk-adapted hybrid model: mandatory dosimetry for high-risk subgroups (CKD, prior myelosuppression, pelvic irradiation), while pragmatically exploring simplified surrogates for routine practice, pending prospective validation of their non-inferiority in therapeutic outcomes. Despite substantial progress, several barriers continue to limit the routine implementation of personalized dosimetry in clinical practice, including the lack of standardized imaging protocols, workflow complexity, limited cost-effectiveness, and insufficient large-scale prospective validation. In addition, individualized dose optimization remains a critical prerequisite for maximizing therapeutic efficacy while minimizing treatment-related toxicity. Reflecting this paradigm shift, the 2023 updated guidelines from the European Association of Nuclear Medicine (EANM) recommend that, whenever feasible, radioligand therapy should incorporate patient-specific dosimetry based on quantitative organ uptake rather than fixed administered activity. Specifically, the guidelines state that “whenever possible, individualized dosing based on organ uptake should be used” and recommend quantitative imaging at a minimum of two time points—an early acquisition (approximately 4 h) and a delayed acquisition (approximately 24 h)—to enable reliable absorbed dose estimation (Kratochwil et al., 2023). Similarly, the 2024 consensus statement from the American College of Radiology (ACR) emphasizes that dose individualization is fundamental to precision radiopharmaceutical therapy rather than an optional refinement (Wallner et al., 2024). Ongoing prospective clinical trials, together with advances in rapid quantitative imaging, artificial intelligence-assisted dosimetry, automated image analysis, and clinical decision-support systems, are expected to accelerate the development of a standardized, clinically applicable, and evidence-based framework for personalized dosimetry in radiopharmaceutical therapy. Third, limitations in clinical evidence and trial design hinder the robust evaluation and optimal positioning of ^177^Lu-based therapies (Schnog et al., 2024; Viscuse et al., 2024). Future progress requires biomarker-driven, randomised clinical trials with harmonised endpoints, alongside adaptive trial designs that integrate dosimetry and translational biomarkers to generate more reproducible and generalisable evidence. Fourth, the emergence of treatment resistance poses a growing challenge to the durability of response (Arbuznikova et al., 2023; Waldeck et al., 2023). Acquired resistance to ^177^Lu-based radiopharmaceutical therapy is multifactorial and remains a major challenge to durable clinical benefit. Resistance arises not only from reduced target expression or antigen loss but also from enhanced DNA double-strand break repair through the non-homologous end joining (NHEJ) and homologous recombination (HR) pathways, intratumoral heterogeneity that enables the emergence of resistant subclones, and an immunosuppressive tumor microenvironment (TME). The latter is characterized by HIF-1α-driven hypoxia and paracrine signaling mediated by cancer-associated fibroblasts (CAFs) and myeloid-derived suppressor cells (MDSCs), which collectively attenuate radiation-induced immunogenic cell death and foster a therapy-resistant niche. To overcome these mechanisms, several rational combination strategies have shown encouraging preclinical activity, including PARP inhibitors to disrupt DNA damage repair, immune checkpoint inhibitors to restore antitumor immunity and remodel the TME, and heterobivalent radioligands designed to overcome target heterogeneity and antigen loss. Nevertheless, the efficacy, safety, and therapeutic index of these combination approaches remain to be established through well-designed prospective clinical trials across diverse patient populations.

Fifth, isotope supplication, infrastructure and workforce limitations continue to restrict global accessibility (Veit-Haibach et al., 2025).

The health-economic sustainability of ^177^Lu radiopharmaceutical therapy depends on balancing its substantial lifecycle costs against its demonstrated clinical benefits. Total costs extend well beyond radiopharmaceutical production, encompassing isotope manufacturing based on limited reactor capacity and enriched target materials, investment in nuclear medicine infrastructure, radiation safety and waste management, specialized workforce training, and indirect costs incurred by patients. Economic evaluations based on the VISION trial indicate that, despite meaningful improvements in clinical outcomes, the incremental cost-effectiveness ratio (ICER) of ^177^Lu-PSMA therapy for metastatic castration-resistant prostate cancer (mCRPC) frequently exceeds the willingness-to-pay thresholds adopted in many healthcare systems, highlighting the need for optimized patient selection and value-based reimbursement strategies. The dependence on reactor-based ^177^Lu production further exposes vulnerabilities in the global supply chain. Limited production capacity, reliance on highly enriched target materials, and radionuclidic impurities such as ^177^mLu not only constrain isotope availability but also increase the complexity of regulatory approval, radioactive waste management, and international distribution (Mathur et al., 2017). These challenges are compounded by fragmented regulatory oversight, the absence of harmonized standards for clinical development, manufacturing, dosimetry, and post-marketing surveillance, as well as logistical barriers associated with transporting short-lived radioactive products across international borders. Expanding isotope production capacity, improving GMP-compliant automated radiolabeling technologies (Li et al., 2024), and harmonizing international regulatory frameworks will be critical to ensuring a reliable and sustainable supply chain.

Equitable access to ^177^Lu radiopharmaceutical therapy also varies substantially across healthcare systems. In high-income countries, ^177^Lu-DOTATATE has been widely incorporated into reimbursement programs for neuroendocrine tumors, whereas access to ^177^Lu-PSMA therapy remains restricted in many jurisdictions because of reimbursement, infrastructure, and capacity limitations. Middle-income countries have made progress toward domestic or regional isotope production; however, treatment availability remains constrained by the uneven distribution of nuclear medicine facilities and trained personnel. In contrast, most low-income countries lack the infrastructure required for radiopharmaceutical therapy, including isotope production capability, radiopharmacy services, radiation safety systems, and specialized clinical expertise. Consequently, even when radiopharmaceuticals are available through international assistance programs, implementation is often limited by the substantial investment required for facility development, regulatory compliance, and workforce training. Addressing these disparities will require coordinated investment in infrastructure, multidisciplinary workforce development, technology transfer, and international collaboration to promote equitable global access to ^177^Lu radiopharmaceutical therapy (Arbuznikova et al., 2024; Ninatti et al., 2025). To accelerate the clinical evolution of ^177^Lu-based radiopharmaceutical therapy, we propose a three-tiered roadmap encompassing fundamental scientific questions, clinical priorities, and translational implementation over the next decade. At the fundamental level, several critical knowledge gaps remain. First, radiopharmaceutical therapy (RLT)-specific organ dose constraints require prospective validation, as the currently accepted kidney (23 Gy) and bone marrow (2 Gy) limits are largely extrapolated from external beam radiotherapy (EBRT) rather than derived from radiobiological data specific to radionuclide therapy. Second, the molecular basis of treatment resistance remains incompletely understood and extends beyond target downregulation to encompass alterations in DNA damage response pathways, intratumoral heterogeneity, and the immunosuppressive tumor microenvironment. Third, robust predictive biomarkers and dosimetric criteria are needed to guide patient selection and determine the optimal application of β-versus α-emitting radionuclides. Clinically, the field is shifting from late-line salvage therapy toward earlier disease stages and mechanism-based combination strategies. Multiple prospective trials are evaluating ^177^Lu-PSMA in combination with external beam radiotherapy, while additional ligand-targeted radiopharmaceuticals directed against fibroblast activation protein (FAP), gastrin-releasing peptide receptor (GRPR), and CXC chemokine receptor 4 (CXCR4) are advancing through clinical development. At the same time, α-emitting radiopharmaceuticals, particularly ^225^Ac-based therapies, are emerging as promising treatment options for patients with resistance to β-emitting radionuclides, although their relative clinical value and optimal sequencing remain to be established in randomized studies. From a translational perspective, future progress will depend on integrating patient-specific dosimetry into routine clinical practice through artificial intelligence-assisted image analysis and automated treatment planning, developing biologically rational combination regimens incorporating DNA damage response inhibitors and immunotherapy, engineering next-generation ligands with improved tumor specificity and pharmacokinetic properties, and expanding isotope production and GMP-compliant radiopharmaceutical manufacturing. Harmonized regulatory frameworks, standardized dosimetry methodologies, and international multicenter collaborations will be equally important for generating high-quality evidence and accelerating global implementation.

**FIGURE 7:**
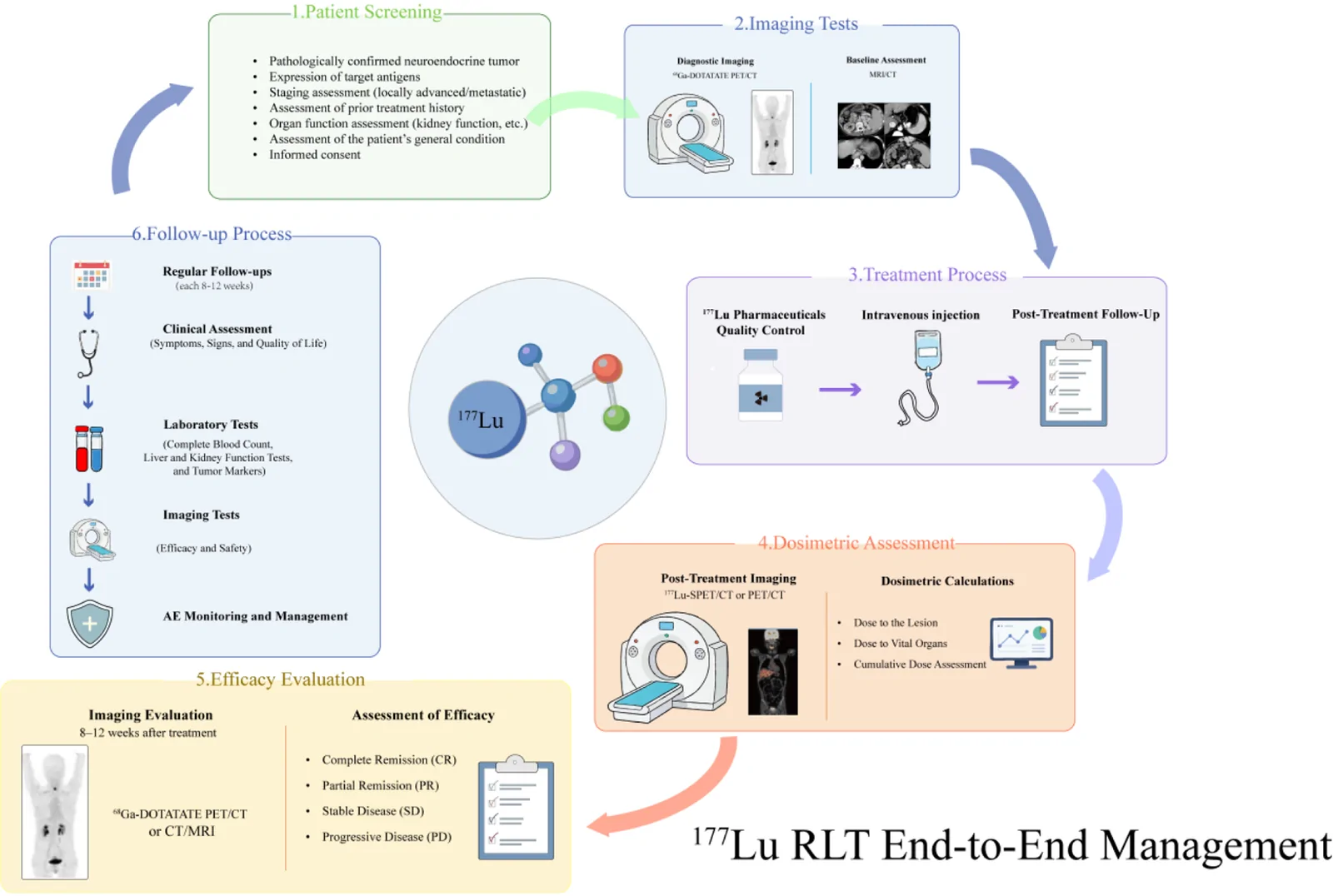
Comprehensive management algorithm for ^177^Lu radioligand therapy:This figure illustrates the comprehensive closed-loop management pathway for ^177^Lu radioligand therapy (RLT) in patients with SSTR-positive neuroendocrine tumors (NETs), encompassing the entire continuum from patient screening to long-term follow-up. The workflow commences with pathologically confirmed NETs and diagnostic ^68^Ga-DOTATATE PET/CT for target expression assessment, followed by baseline multi-dimensional evaluation (staging, prior treatment history, organ function, ECOG performance status) to identify eligible candidates. The treatment phase comprises intravenous administration of ^177^Lu-labeled ligand, quality control, post-administration monitoring, and nursing guidance. Post-therapy, individualized dosimetry is performed using ^177^Lu SPECT/CT, including tumor-absorbed dose, cumulative dose, and organs-at-risk (kidneys, bone marrow) dose calculations, to ensure an optimal efficacy-safety balance. Follow-up assessments are conducted at 8–12 weeks intervals, incorporating clinical evaluation, laboratory tests, and imaging surveillance via ^68^Ga-DOTATATE PET/CT or CT/MRI. Response is categorized according to RECIST 1.1 or PERCIST criteria, integrated with clinical symptoms and tumor biomarkers. The entire process is coordinated through multidisciplinary team (MDT) collaboration involving nuclear medicine, oncology, radiology, medical physics, pharmacy, and nursing, with the ultimate goals of prolonging survival, alleviating symptoms, and improving quality of life, thereby enabling safe and individualized therapy.

Collectively, these priorities define a pathway toward precision radiopharmaceutical therapy, in which biologically informed patient selection, individualized dosimetry, rational therapeutic combinations, and coordinated infrastructure development converge to maximize clinical benefit while ensuring sustainable and equitable access to ^177^Lu-based treatment worldwide. In addition to the well-established adverse reactions of acute hematologic toxicity and mild dry mouth, adverse reactions associated with the clinical use of ^177^Lu-labeled radiopharmaceuticals also include secondary malignancies, cumulative bone marrow toxicity, and progressive renal impairment. Although the risk of secondary malignancies is relatively low in the general population (5–10-year incidence of 1%–2%), this risk cannot be ignored in younger patients (<65 years of age) or those with DNA damage response (DDR) gene abnormalities; treatment-related myelodysplastic syndrome (MDS) and acute myeloid leukemia (AML) have a latency period of 2–7 years (George and Samuel, 2023). Cumulative bone marrow toxicity manifests as progressive decline in hematopoietic reserve, particularly refractory thrombocytopenia and anemia that persist beyond 12 months of treatment; this is most commonly observed in patients whose bone marrow has already been severely compromised by bone metastases or prior cytotoxic therapy. Although renal impairment rarely progresses to end-stage renal failure under current renal protection regimens, patients experience a slight but persistent decline in estimated glomerular filtration rate (eGFR) 2–5 years after treatment. To address these delayed toxicities, this article recommends a risk-stratified monitoring strategy: patients who have received ≥4 treatment cycles or have a history of bone marrow toxicity should undergo annual complete blood count follow-up for life; patients with baseline chronic kidney disease or hypertension should have their renal function assessed every 6 months; and given that the drug is primarily excreted via the urinary system, low-threshold screening for bladder and colorectal malignancies should be maintained. There is an urgent need to establish a prospective registry to validate this monitoring strategy and optimize individualized monitoring protocols. In addition, safe clinical implementation necessitates robust radiation protection systems to mitigate risks of external exposure, internal contamination, and environmental dissemination (Herrmann et al., 2022), as well as optimised management of treatment-related toxicities, including those associated with adjunctive therapies such as amino acid infusions (Rolleman et al., 2003; Bodei et al., 2013). Figure 7 illustrates the full-process management of ^177^Lu radioligand therapy, using the treatment of neuroendocrine tumors as an example.

In conclusion, ^177^Lu radiopharmaceuticals have demonstrated their advantages in targeted delivery and sustained irradiation in preclinical models, laying the translational foundation for precision cancer therapy. However, their routine clinical adoption still requires addressing key issues such as standardization of dosimetry, optimization of combination strategies, and extrapolation of toxicity across species. The field is currently at a pivotal juncture, transitioning from proof of concept to evidence-based practice, and there is an urgent need to translate experimental evidence into reproducible clinical benefits through image-guided dose modulation and prospective randomized controlled trials.

As a cornerstone of precision radionuclide therapy, ^177^Lu-based radiopharmaceutical therapy has demonstrated substantial clinical benefit across multiple malignancies and is now incorporated into international evidence-based clinical practice guidelines. Currently, ^177^Lu radiopharmaceuticals are approved for the treatment of somatostatin receptor (SSTR)-positive gastroenteropancreatic neuroendocrine tumors (GEP-NETs), prostate-specific membrane antigen (PSMA)-positive metastatic castration-resistant prostate cancer (mCRPC), and iodine-refractory differentiated thyroid cancer in selected clinical settings. In addition, radionuclide therapies play an established role in the palliation of pain associated with bone metastases.

Despite these advances, several biological and clinical challenges continue to limit treatment efficacy. Inter- and intratumoral heterogeneity in target expression can compromise patient selection and contribute to primary or acquired resistance. Moreover, although the short tissue penetration range of β-particles emitted by ^177^Lu (approximately 1–2 mm) enables localized irradiation and a favorable therapeutic index, radiation exposure to adjacent normal tissues remains an important consideration, particularly in organs at risk. Dose intensification is further constrained by cumulative hematological toxicity, especially bone marrow suppression, and radiation-induced nephrotoxicity, highlighting the need for individualized dosimetry and optimized treatment strategies.

Future development of ^177^Lu radiopharmaceutical therapy is likely to progress along three complementary directions. In the short term (1–3 years), integrating molecular, imaging, and circulating biomarkers into patient stratification is expected to improve treatment selection and response prediction. In the medium term (3–5 years), the development of next-generation ligands with enhanced tumor specificity and improved pharmacokinetic properties, together with artificial intelligence-assisted personalized dosimetry, may further optimize the therapeutic index and reduce treatment-related toxicity. Over the longer term (>5 years), biologically rational combination strategies incorporating immunotherapy, DNA damage response inhibitors, and other targeted therapies are anticipated to transform ^177^Lu radiopharmaceutical therapy from a largely protocol-driven approach into a truly precision medicine paradigm characterized by individualized patient selection, adaptive dosing, and mechanism-based therapeutic combinations.

## Glossary

RLT: Radioligand Therapy
RNT: Radionuclide Therapy
SPECT: Single Photon Emission Computed Tomography
PET: Positron Emission Tomography
NETs: Neuroendocrine Tumors
FDA: Food and Drug Administration
PRRT: Peptide Receptor Radionuclide Therapy
PFS: Progression Free Survival
OS: Overall Survival
rPFS: radiographic Progression Free Survival
BSC: Best Supportive Care
SSTR: Somatostatin Receptor
PSMA: Prostate Specific Membrane Antigen
TXA: Tranexamic Acid
ADC: Antibody-Drug Conjugate
BsAbs: Bispecific Antibodies
oxMIF: oxidized macrophage Migration Inhibitory Factor
HSG: Histamine Succinyl Glycine
FAP: Fibroblast Activation Protein
FAPIs: FAP Inhibitors
CAIX: Carbonic Anhydrase IX
TME: Tumor Microenvironment
GMLs: Glucose-Modified Liposomes
EBRT: External Beam Radiation Therapy
IMRT: Intensity-Modulated Radiotherapy
RIT: Radioimmunotherapy
hK2: human Kallikrein 2
FcRn: Fc Receptor
MAbs: Monoclonal Antibodies
SCLC: Small Cell Lung Cancer
pRIT: pretargeting Radioimmunotherapy
TRT: Targeted Radionuclide Therapy
ADT: Androgen Deprivation Therapy
RTKs: Receptor Tyrosine Kinases
PROTACs: Proteolysis-Targeting Chimeras.
