# Supplementary material for: Lutetium-177 radiopharmaceuticals in cancer care: progress and challenges
Source: Front Pharmacol. 2026 Sep 7;17:1907065. doi: 10.3389/fphar.2026.1907065 (PMC13591410; doi:10.3389/fphar.2026.1907065)
Supplement: Supplementary file 1 [file Table1.docx]

**Table S1** Clinical trials of Lutetium-177 Radiopharmaceuticals in Cancer from clinicaltrials. gov

| **NCT Number** | **Status** | **Phase** | **Cancer Type** | **Stage of Cancer** | **Radiotheraputic agents** | **Outcome Measure** | **Study Aims** | **Sam-ple Size** | **Start Date** |
| --- | --- | --- | --- | --- | --- | --- | --- | --- | --- |
| NCT04801264 | Unknown | Early Phase 1 | Adenoid Cystic Carcinoma | NA | ^177^Lu-EB-PSMA-617 | CTCAE v4. 0 Therapeutic Response | Drug testing | 90 | 2020-09-01 |
| NCT04665947 | Recruiting | Early Phase 1 | Pancreatic Ductal Adenocarcinoma | Advanced/Metastasis | ^177^Lu-DOTA-ABM-5G | DLT DA | Drug testing | 30 | 2020-12-18 |
| NCT05623891 | Unknown status | Early Phase 1 | ED-B-positive Solid tumor | NA | ^177^Lu-B5-IgG4 | AD AE Incidence SUVs | Drug testing | 10 | 2022-12 |
| NCT05603559 | Recruiting | Early Phase 1 | Castration-Resistant Prostate Cancer | Metastasis | ^177^Lu-P17-087/^177^Lu-P17-088 | Dosimetry AE Incidence PSA Response PSMA Response | Drug testing | 30 | 2023-01-01 |
| NCT06050239 | Unknown | Early Phase 1 | Prostate cancer | Metastasis | ^177^Lu-PSMA-0057 | PSA50,PSA90 rPFS | Drug testing | 10 | 2023-03-01 |
| NCT05985278 | Recruiting | Early Phase 1 | Liver Cancer/Breast cancer/Prostate cancer | Advanced | ^177^Lu-Catalase | AD TV AE Incidence | Drug testing | 10 | 2023-07-06 |
| NCT06638034 | Recruiting | Early Phase 1 | FAP-positive cancer/ RGD-positive cancer | Metastasis | ^177^Lu-FAPI-RGD | AE Incidence SUVs | Dosimetry | 15 | 2024-05-01 |
| NCT06305962 | Recruiting | Early Phase 1 | Solid tumor | Metastasis | ^177^Lu-RAD204 | TAC AD TIAC AE Incidence DLT | Drug testing/  Radiation dosimetry | 23 | 2024-06-03 |
| NCT06546527 | Not yet recruiting | Early Phase 1 | Castration-Resistant Prostate Cancer | Metastasis | ^177^Lu-PSMA-137 | AE Incidence | Drug testing/  Radiation dosimetry | 12 | 2024-08-01 |
| NCT06636617 | Recruiting | Early Phase 1 | FAP-positive cancer | NA | ^177^Lu-JH04 | Dosimetry TV | Drug testing/  Radiation dosimetry | 9 | 2024-08-21 |
| NCT06632873 | Recruiting | Early Phase 1 | SSTR2-positive cancer/αVβ3-positive cancer | NA | ^177^Lu-TATE-RGD | AE Incidence SUVs | Drug testing | 10 | 2024-09-01 |
| NCT06639191 | Not yet recruiting | Early Phase 1 | CD44v6-positive Solid tumor | Advanced/Metastasis | ^177^Lu-AKIR001 | DLT RP2D AE Incidence AD DoR DCR | Drug testing | 15 | 2024-11-01 |
| NCT06824155 | Recruiting | Early Phase 1 | HER2-positive Solid tumor | Advanced | ^177^Lu-RAD202 | TAC AD AE Incidence DLTs ORR TIAC Blood half-life | Drug testing/  Radiation dosimetry | 35 | 2025-02-12 |
| NCT06898437 | Recruiting | Early Phase 1 | FAP-positive Radioactive Iodine-Refractory Differentiated Thyroid Cancer | NA | ^177^Lu-LNC1004 | ORR DCR DoR PFS BR AE | Drug testing | 20 | 2025-03-24 |
| NCT07229768 | Recruiting | Early Phase 1 | Solid tumor | Advanced | ^177^Lu-CTR-FAPI | AE Radiation Dose ORR | Drug testing | 4 | 2025-10-15 |
| NCT05613738 | Recruiting | NA | Castration-Resistant Prostate Cancer | Metastasis | ^177^Lu-PSMA-EB-01 | Dosimetry AE Incidence PSA Response | Drug testing | 9 | 2022-11-23 |
| NCT05644080 | Recruiting | NA | Glioma | Recurrent | ^177^Lu-PSMA I&T | AE Incidence AD OS、PFS | Drug testing | 10 | 2023-03-28 |
| NCT065^177^19 | Recruiting | NA | Castration-Resistant Prostate Cancer | Metastasis | ^177^Lu-vipivotide tetraxetan | FACT-P FACT-RNT BPI-SF PFS OS | Clinical practice | 500 | 2024-09-04 |
| NCT07014254 | Not yet recruiting | NA | Gastrointestinal malignant tumor | Advanced/Metastasis | ^177^Lu-CTR-FAPI | ORR DCR | Drug testing/ Radiation dosimetry | 20 | 2025-06-20 |
| NCT05053854 | Recruiting | Phase 1 | Gastroenteropancreatic Neuroendocrine Tumor | Metastasis | ^177^Lu-DOTA-Octreotate with talazoparib | MTD DLT AE rPFS OS | Combined treatment | 24 | 2021-12-08 |
| NCT04786847 | Completed | Phase 1 | Castration-Resistant Prostate Cancer | Metastasis | ^177^Lu-DOTA-TLX591 | AD AE | Drug testing | 30 | 2022-01-30 |
| NCT04375267 | Active, not recruiting | Phase 1 | SSTR-positive Neuroendocrine tumor | Metastasis | ^177^Lu-DOTATATE with olaparib | AE Incidence DoR TTP | NA | 18 | 2022-04-23 |
| NCT05109728 | Recruiting | Phase 1 | Glioblastoma | Recurrent | ^177^Lu-DOTA-TATE with Temozolomide | DLTs AD AEs PFS、OS TAC PK Parameters | Combined treatment/ Dosimetry | 60 | 2022-05-10 |
| NCT05475210 | Recruiting | Phase 1 | SSTR-positive Neuroendocrine tumor | Advanced | ^177^Lu-DOTA-EB-TATE | AE Incidence DLT MTD | Drug testing/ Dosimetry | 9 | 2022-06-18 |
| NCT05410821 | Completed | Phase 1 | Radioactive Iodine-Refractory Differentiated Thyroid Cancer | Metastasis | ^177^Lu-DOTA-EB-FAPI | AE Incidence MTD  ORR AD | Drug testing/ Dosimetry | 12 | 2022-06-21 |
| NCT03966651 | Recruiting | Phase 1 | Neuroblastoma | Recurrent | ^177^Lu-DOTA0-Tyr3-octerotate | AE Incidence MTD | Drug testing | 18 | 2023-04-17 |
| NCT05868174 | Recruiting | Phase 1 | CAIX-positive Solid tumor | Advanced/Metastasis | ^177^Lu-TLX250 with Peposertib | DLT AE Incidence OS、PFS ORR | Combined treatment/ Dosimetry | 36 | 2023-05-23 |
| NCT05613894 | Recruiting | Phase 1 | Castration-Resistant Prostate Cancer | Metastasis | ^177^Lu-PSMA-617 with Cabozantinib | DLT PFS,OS,rPFS) ORR,DoR PSA50 | Combined treatment/ Efficacy Comparison | 33 | 2023-07-14 |
| NCT06081322 | Unknown | Phase 1 | Pancreatic Cancer/Cholangiocarcinoma | Advanced | ^177^Lu-EB-FAPI | PFS,OS ORR,DOR,DCR | Drug testing | 29 | 2023-09-01 |
| NCT05723640 | Completed | Phase 1 | FAP-positive Solid tumor | Advanced/Metastasis | ^177^Lu-LNC1004 | AE Incidence DLT | Drug testing | 11 | 2023-10-03 |
| NCT05870579 | Recruiting | Phase 1 | Breast cancer | Advanced | ^177^Lu-NeoB with ribociclib & fulvestrant | DLT AE,SAEs TAC Radiation Dose PK Parameters ORR、DoR、TTR PFS、OS | Combined treatment | 48 | 2023-11-13 |
| NCT06197139 | Recruiting | Phase 1 | FAP-positive Solid tumor | Advanced | ^177^Lu-XT117 | AE Incidence ORR,DoR,DCR PFS,OS | Drug testing | 20 | 2024-01 |
| NCT06228482 | Recruiting | Phase 1 | Non-small cell lung cancer | Metastasis | ^177^Lu-DOTA-ABM-5G | ORR,DoR | Efficacy Assessment | 40 | 2024-01-22 |
| NCT06303713 | Recruiting | Phase 1 | Castration-Resistant Prostate Cancer | Metastasis | ^177^Lu-PSMA-617 with carboplatin | MTD,RP2D PSA Response ORR rPFS,OS | Combined treatment | 37 | 2024-05-22 |
| NCT06375564 | Recruiting | Phase 1 | Solid tumor | Advanced | ^177^Lu-AB-3PRGD2 | ORR AE Incidence | Drug testing | 20 | 2024-06-12 |
| NCT06553846 | Not yet recruiting | Phase 1 | Solid tumor | Advanced/Metastasis | ^177^Lu-FAP-75 | ORR,DCR,DoR AE DLT OS,PFS | Drug testing/  Radiation dosimetry | 50 | 2024-08-19 |
| NCT07096128 | Recruiting | Phase 1 | Prostate cancer | Metastasis | ^177^Lu-PSMA-XT | AE Incidence DLT AD PSA50 rPFS ORR,DoR,DCR SSE | Drug testing/  Radiation dosimetry | 36 | 2024-08-20 |
| NCT06641219 | Completed | Phase 1 | Prostate cancer | NA | ^177^Lu-BQ7876 | SUVmean AE Incidence | Drug testing/ Dosimetry | 7 | 2024-10-01 |
| NCT06562192 | Recruiting | Phase 1 | Pancreatic Cancer/lung cancer/Breast cancer/Colorectal carcinoma | Advanced/Metastasis | ^177^Lu-NNS309 | DLT AD AE PFS ORR,DoR,DCR PK Parameters | Drug testing/ Dosimetry | 124 | 2024-10-15 |
| NCT06649682 | Recruiting | Phase 1 | Clear cell renal cell carcinoma | Metastasis | ^177^Lu-NYM096 | AE Incidence DLT PFS,OS ORR,DCR,DoR | Efficacy Assessment | 20 | 2024-11-21 |
| NCT06663072 | Recruiting | Phase 1 | Pancreatic Neuroendocrine tumor | Advanced | ^177^Lu-DOTATATE with Fulvestrant | ORR PFS、OS | Efficacy Assessment | 25 | 2024-12-13 |
| NCT06237491 | Not yet recruiting | Phase 1 | Castration-Resistant Prostate Cancer | Metastasis | ^177^Lu-LNC1003 | AE Incidence DLT MTD | Drug testing/ Dosimetry | 12 | 2025-05-07 |
| NCT06640413 | Not yet recruiting | Phase 1 | FAP-positive Solid tumor | Advanced/Metastasis | ^177^Lu-OncoFAP-23 | AEs,SAEs DLT | Combined treatment/ Dosimetry | 56 | 2025-05-31 |
| NCT06911489 | Not yet recruiting | Phase 1 | FAP-positive Solid tumor | Advanced | ^177^Lu-NRT6020 | AE Incidence DLT RP2D SUV AD PFS ORR,DOR PK Parameters | Drug testing/ Dosimetry | 116 | 2025-07 |
| NCT07121244 | Recruiting | Phase 1 | Breast cancer | Metastasis/Recurrent | ^177^Lu-R11228 | AEs,SAEs DLT AD PK Parameters ORR,DoR,DCR PFS、OS | Dosimetry | 62 | 2025-10-01 |
| NCT07081555 | Recruiting | Phase 1 | HER2-positive Breast cancer | Metastasis | ^177^Lu-ABY-271 | AE DLT AD Normalized Whole-Body Effective Dose | Drug testing | 21 | 2025-10-06 |
| NCT07090369 | Not yet recruiting | Phase 1 | Castration-Resistant Prostate Cancer | Metastasis | ^177^Lu-PSMA-I&T with Olaparib & Pembrolizumab | ORR PFS,OS PSA50,PSA90 | Efficacy Assessment | 20 | 2025-11-01 |
| NCT07232407 | Not yet recruiting | Phase 1 | Gastrointestinal and other solid tumors | Advanced/Metastasis | ^177^LuBL-ARC001 | DLT MTD RP2D TRAE PK Parameters ORR,DoR,DCR | Drug testing | 22 | 2025-12 |
| NCT07244393 | Not yet recruiting | Phase 1 | Castration-Resistant Prostate Cancer | Metastasis | ^177^Lu-DGUL With Pembrolizumab | AE DLT rPFS PK Parameters ORR,DoR,DCR PSA Response | Drug testing | 30 | 2025-12 |
| NCT06941480 | Not yet recruiting | Phase 1 | Small cell lung cancer/Neuroendocrine prostate cancer | NA | ^177^Lu-DTPA-SC16. 56 | MTD | Efficacy Assessment | 12 | 2026-01 |
| NCT04430192 | Active, not recruiting | Phase 1&Phase 2 | Prostate cancer | Advanced | ^177^Lu-PSMA-617 | AD AE QoL BCR Time | Efficacy Assessment | 20 | 2020-08-06 |
| NCT04997317 | Recruiting | Phase 1&Phase 2 | Meningioma | Recurrent | ^177^Lu-DOTA-JR11&^177^Lu-DOTATOC | Tumor-to-DLO Tumor-to-nDLO PK Parameters MTD AE PFS | Efficacy Assessment | 18 | 2021-04-21 |
| NCT04939610 | Recruiting | Phase 1&Phase 2 | Solid tumor | Advanced/Metastasis | ^177^Lu-FAP-2286 | DLTs,AD MTD SUVmax AE ORR,DoR,DCR PFS PK Parameters | Efficacy Assessment | 222 | 2021-07-30 |
| NCT05410743 | Completed | Phase 1&Phase 2 | SSTR2-positive cancer | NA | ^177^Lu-TATE-EB-01（LNC1010） | AE ORR PFS | Efficacy Assessment | 30 | 2022-06-01 |
| NCT05142696 | Recruiting | Phase 1&Phase 2 | Small cell lung cancer | NA | ^177^Lu-DOTA-TATE with carboplatin, etoposide and atezolizumab | AE DLT OS,PFS ORR,DoR,DCR PK Parameters | Combined treatment | 200 | 2022-07-13 |
| NCT05340374 | Active, not recruiting | Phase 1&Phase 2 | Castration-Resistant Prostate Cancer | Metastasis | ^177^Lu-PSMA-617 with Cabazitaxel | DLT MTD RP2D  AE PSA Response ORR OS,rPFS | Drug testing | 35 | 2022-07-14 |
| NCT05706129 | Recruiting | Phase 1&Phase 2 | Solid tumor | Advanced/Metastasis | ^177^Lu-DPI-4452 | AE DLT ORR,DoR,DCR PFS PK Parameters | Efficacy Assessment | 270 | 2023-03-14 |
| NCT06250244 | Recruiting | Phase 1&Phase 2 | Castration-Resistant Prostate Cancer | Metastasis | ^177^Lu-LNC1011 | Semi-quantitative Dosimetry AE AD | Dosimetry | 12 | 2023-05-01 |
| NCT06081686 | Recruiting | Phase 1&Phase 2 | Prostate cancer | Metastasis | ^177^Lu-XT033 | AE AD PK Parameters PSA50 rPFS ORR,DoR,DCR SSE | Drug testing/  Radiation dosimetry | 32 | 2023-09-13 |
| NCT06184035 | Recruiting | Phase 1&Phase 2 | Solid tumor | Advanced/Recurrent/Metastasis | ^177^Lu-SN201 | AE DLT,MTD,RP2D PK Parameters ORR,DoR,DCR OS,PFS | Drug testing /Dosimetry | 90 | 2023-12-06 |
| NCT06066437 | Recruiting | Phase 1&Phase 2 | Prostate cancer | NA | ^177^Lu-rhPSMA-10. 1 | AE Radiation Dose | Drug testing | 36 | 2024-03-29 |
| NCT06383052 | Active, not recruiting | Phase 1&Phase 2 | Castration-Resistant Prostate Cancer | Metastasis | ^177^Lu-NYM032 | DLT MTD | Drug testing/  Radiation dosimetry | 30 | 2024-04-26 |
| NCT06247995 | Recruiting | Phase 1&Phase 2 | Breast cancer | Metastasis | ^177^Lu-NeoB with Capecitabine | DLTs AE ORR,TTR,DoR OS,PFS、TAC | Efficacy Assessment/ Dosimetry | 58 | 2024-08-14 |
| NCT07189871 | Not yet recruiting | Phase 1&Phase 2 | Solid tumor | Advanced/Recurrent/Metastasis | ^177^Lu-BetaBart | DLTs,AD AE ORR PK Parameters | Efficacy Assessment | 61 | 2025-11 |
| NCT06991738 | Not yet recruiting | Phase 1&Phase 2 | Hurthle cell thyroid carcinoma | Metastasis | ^177^Lu-DOTA-EB-TATE | DLTs AD PFS ORR,DCR AE | Drug testing | 18 | 2025-12-03 |
| NCT04343885 | Active, not recruiting | Phase 2 | Hormone-Naive Prostate Cancer | Metastasis | ^177^Lu-PSMA-617 with Docetaxel | AE PSA-PFS,rPFS,OS | Combined treatment/ Efficacy Comparison | 130 | 2020-04-21 |
| NCT04525638 | Unknown status | Phase 2 | Neuroendocrine tumor | Advanced/Metastasis | ^177^Lu-DOTATATE with Nivolumab | ORR  OS,PFS | Combined treatment | 30 | 2020-06-29 |
| NCT04903899 | Recruiting | Phase 2 | neuroblastoma | Recurrent | ^177^Lu-DOTATATE | AE PFS、OS AD | Efficacy Assessment | 24 | 2021-05-19 |
| NCT04663997 | Active, not recruiting | Phase 2 | Castration-Resistant Prostate Cancer | Metastasis | ^177^Lu-PSMA-617 with Docetaxel | PFS、OS CBR AE | Efficacy Assessment | 200 | 2021-08-11 |
| NCT05239533 | Recruiting | Phase 2 | Renal carcinoma | Advanced | ^177^Lu-girentuximab with Nivolumab | MTD ORR | Combined treatment | 41 | 2022-02-16 |
| NCT05963386 | Completed | Phase 2 | Multiple solid tumors | Advanced/Metastasis | ^177^Lu-DOTA-EB-FAPI | PFS,OS Dosimetry | Efficacy Assessment | 28 | 2022-02-22 |
| NCT05150236 | Active, not recruiting | Phase 2 | Castration-Resistant Prostate Cancer | Metastasis | ^177^Lu-PSMA with Ipilimumab and Nivolumab | AE OS,rPFS, ORR,DoR HRQoL | Combined treatment | 93 | 2022-04-29 |
| NCT05579184 | Active, not recruiting | Phase 2 | Castration-Resistant Prostate Cancer | Metastasis | ^177^Lu-Ludotadipep | PSA-RR SUV ORR PFS | Drug testing | 20 | 2022-10-30 |
| NCT05198479 | Unknown | Phase 2 | Nasopharyngeal carcinoma | Metastasis | ^177^Lu-DOTA0-Tyr3-Octreotate | OS,PFS ORR,TTP | Efficacy Assessment | 25 | 2023-05-05 |
| NCT05894486 | Not yet recruiting | Phase 2 | SSTR-positive Gastroenteropancreatic Neuroendocrine Tumor | Advanced/Metastasis | ^177^Lu-DOTA0-Tyr3-Octreotate | ORR,DCR AE PFS | Drug testing | 40 | 2023-06-05 |
| NCT05560659 | Recruiting | Phase 2 | Prostate cancer | Advanced/Metastasis | ^177^Lu-PSMA with SABR | AE PSA Response OS,bPFS | Efficacy Comparison | 92 | 2023-12-14 |
| NCT05766371 | Recruiting | Phase 2 | Castration-Resistant Prostate Cancer | Metastasis | ^177^Lu-PSMA-617 with Pembrolizumab | OS,rPFS,PSA-PFS ORR,DoR PSA50,PSA90 | Efficacy Assessment | 48 | 2023-12-15 |
| NCT05849298 | Recruiting | Phase 2 | PSMA-positive Castration-Resistant Prostate Cancer | NA | ^177^Lu-Vipivotide Tetraxetan With ARPI | MFS,rPFS,OS SSE FACT-P FACT-RNT | Combined treatment | 80 | 2024-01-03 |
| NCT06607692 | Recruiting | Phase 2 | SSTR-positive Solid tumor | Recurrent | ^177^Lu-DOTATATE with Olaparib | ORR | Efficacy Assessment | 25 | 2024-10 |
| NCT06449781 | Recruiting | Phase 2 | Prostate cancer | NA | ^177^Lu-vipivotide tetraxetan | bPFS,rPFS AE QoL | Efficacy Assessment | 200 | 2025-01-09 |
| NCT06322576 | Recruiting | Phase 2 | Adenoid Cystic Carcinoma | Recurrent/Metastasis | ^177^Lu-PNT2002 | AD ORR,DoR OS,PFS QoL | Drug testing | 10 | 2025-02-10 |
| NCT06880757 | Not yet recruiting | Phase 2 | bladder cancer | Advanced | ^177^Lu-FAP-2286 | AE Radiation Dose ORR | Efficacy Assessment | 10 | 2025-06-01 |
| NCT07047118 | Recruiting | Phase 2 | Castration-Resistant Prostate Cancer | Metastasis | ^177^Lu-Vipivotide Tetraxetan with JSB462 (Luxdegalutamide) | PSA30,PSA50,PSA90 SSE OS,rPFS ORR,DCR,DoR,TTR PK Parameters AD | Combined treatment | 130 | 2025-07-03 |
| NCT06964958 | Not yet recruiting | Phase 2 | PSMA-positive Renal cell carcinoma | Advanced | ^177^Lu-PSMA-617 | ORR AE OS,PFS | Drug testing | 24 | 2025-11 |
| NCT06955169 | Not yet recruiting | Phase 2 | Intracranial meningioma | NA | ^177^Lu-DOTATATE | PFS、OS DCR、ORR TRAE | Efficacy Assessment | 153 | 2025-11-30 |
| NCT04529044 | Not yet recruiting | Phase 2 | Breast cancer | Recurrent | ^177^Lu-DOTATATE | ORR,DCR,DoR PFS AE | Efficacy Assessment | 10 | 2025-12-01 |
| NCT06398444 | Active, not recruiting | Phase 2&Phase 3 | SSTR-positive Neuroendocrine tumor | Advanced/Metastasis | ^177^Lu-Oxodotreotide | AE ORR,DCR,DoR,TTR OS,PFS QoL | Drug testing | 74 | 2024-06-11 |
| NCT04647526 | Active, not recruiting | Phase 3 | Castration-Resistant Prostate Cancer | Metastasis | ^177^Lu-PNT2002 | OS,rPFS,bPFS ORR,DoR PSA Response | Efficacy Assessment | 415 | 2021-02-25 |
| NCT04720157 | Active, not recruiting | Phase 3 | Hormone-Naive Prostate Cancer | Metastasis | ^177^Lu-PSMA-617 with Standard of Care | OS,PFS,rPFS PSA90 SSE ORR,DoR,DCR,TTR FACT-P、PBI-SF、EQ-5D-5L AE | Combined treatment/ Efficacy Comparison | 1145 | 2021-06-09 |
| NCT04919226 | Active, not recruiting | Phase 3 | SSTR-positive Gastroenteropancreatic Neuroendocrine Tumor | NA | ^177^Lu-edotreotide | PFS、OS | Drug testing | 259 | 2021-12-21 |
| NCT05204927 | Active, not recruiting | Phase 3 | Castration-Resistant Prostate Cancer | Metastasis | ^177^Lu-PSMA-I&T VS Abiraterone with Prednisone/Enzalutamide | OS,rPFS PSA50 SSE QoL | Efficacy Assessment | 439 | 2022-02-14 |
| NCT05459844 | Active, not recruiting | Phase 3 | SSTR-positive Gastroenteropancreatic Neuroendocrine Tumor | Advanced/Metastasis | ^177^Lu-Oxodotreotide VS  Octreotide LAR | OS,PFS ORR、DoR、DCR、TTR QoL AE | Efficacy Comparison | 196 | 2022-08-31 |
| NCT05387603 | Recruiting | Phase 3 | Neuroendocrine tumor | Advanced | ^177^Lu-DOTATOC with Capecitabine | OS,PFS AE AD QoL | Efficacy Comparison | 300 | 2022-11-01 |
| NCT05884255 | Recruiting | Phase 3 | SSTR-positive Gastroenteropancreatic Neuroendocrine Tumor | Advanced | ^177^Lu-Oxodotreotide with Standard-dose Long-acting Octreotide | OS,PFS ORR,DoR,DCR AE,SAE | Combined treatment | 220 | 2023-07-06 |
| NCT04876651 | Recruiting | Phase 3 | Castration-Resistant Prostate Cancer | Metastasis | ^177^Lu-DOTA-  rosopatamb | SSE OS,PFS,rPFS ORR Radiation Dose PK Parameters | Efficacy Comparison | 392 | 2023-08-29 |
| NCT05918302 | Recruiting | Phase 3 | SSTR-positive Pulmonary/Thymic Neuroendocrine Tumor | Advanced | ^177^Lu-edotreotide | PFS、OS、ORR、DoR、QoL | Drug testing | 120 | 2023-10-27 |
| NCT06520345 | Recruiting | Phase 3 | Castration-Resistant Prostate Cancer | Metastasis | ^177^Lu-TLX591 with SOC | rPFS、OS ORR SSE | Combined treatment/ Efficacy Comparison | 430 | 2024-07-26 |
| NCT06496581 | Recruiting | Phase 3 | Hormone-Naive Prostate Cancer | Metastasis | ^177^Lu-PSMA-617 with Standard of Care | OS、rPFS PCSS | Combined treatment | 500 | 2024-09-12 |
| NCT06784752 | Recruiting | Phase 3 | Gastroenteropancreatic Neuroendocrine Tumor | Advanced | ^177^Lu-DOTA-TATE with octreotide LAR | OS,PFS ORR,DoR,DCR | Drug testing | 240 | 2025-05-30 |
| NCT06878664 | Recruiting | Phase 3 | Gastroenteropancreatic Neuroendocrine Tumor | Advanced/Metastasis | ^177^Lu-Dotatate | OS,PFS ORR,DoR,DCR AE | Drug testing | 166 | 2025-06-12 |
| NCT07197580 | Not yet recruiting | Phase 3 | Clear cell renal cell carcinoma | Advanced/Recurrent | ^177^Lu-TLX250 | AE OS,mPFS ORR,DCR AD | Drug testing | 40 | 2025-11-15 |

Note: DLT:Dose Limiting Toxicity DA:Dose Assessment AE Incidence:Adverse Event Incidence SUVs:Standardized Uptake Value PSA:Prostate Specific Antigen PSMA:Prostate Specific Membrane Antigen TV:Tumor Volume TAC:Time-Activity Curve TIAC:Time-Integrated Activity Coefficient RP2D:Recommended Phase II Dose DOR:Duration of Response DCR:Disease Control Rate ORR: Objective response rate PFS: Progression-free survival BR:Biochemical Response OS: Overall survival. MTD:Maximum Tolerated Dose rPFS:Radiation Progression-Free Survival TTP:Time to Progression PK:Pharmacokinetic MTA:Maximum Tolerated Activity SSE:Symptomatic Skeletal Event TRAE:Treatment-Related Adverse Event BCR:Biochemical Recurrence DLO:Dose-Limiting Organ CBR:Clinical Benefit Rate MFS:Metastasis-Free Survival PCSS:Prostate Cancer–Specific Survival AD:Absorbed dose
